# *Yersinia enterocolitica* YopT and *Clostridium difficile* Toxin B Induce Expression of GILZ in Epithelial Cells

**DOI:** 10.1371/journal.pone.0040730

**Published:** 2012-07-09

**Authors:** Martin Köberle, David Göppel, Tanja Grandl, Peer Gaentzsch, Birgit Manncke, Susanne Berchtold, Steffen Müller, Bernhard Lüscher, Marie-Liesse Asselin-Labat, Marc Pallardy, Isabel Sorg, Simon Langer, Holger Barth, Robert Zumbihl, Ingo B. Autenrieth, Erwin Bohn

**Affiliations:** 1 Interfaculty Institute of Microbiology and Infection Medicine, Eberhard Karls University of Tübingen, Tübingen, Germany; 2 Dermatology, Eberhard Karls University of Tübingen, Tübingen, Germany; 3 Institut für Biochemie und Molekularbiologie, Universitätsklinikum RWTH Aachen, Aachen, Germany; 4 Universud, NSERM UMR-S 996, Faculte de Pharmacie Paris-Sud, Chatenay-Malabry, France; 5 The Walter and Eliza Hall Institute of Medical Research, Parkville, Melbourne, Australia; 6 Biozentrum der Universität Basel, Basel, Switzerland; 7 Institute of Pharmacology and Toxicology, University of Ulm Medical Center, Ulm, Germany; 8 INRA, UMR1333, Laboratoire Diversité, Génomes et Interactions Microorganismes Insectes, Montpellier, France; 9 Université de Montpellier 2, Montpellier, France; Universidad de Costa Rica, Costa Rica

## Abstract

Glucocorticoid induced-leucine zipper (GILZ) has been shown to be induced in cells by different stimuli such as glucocorticoids, IL-10 or deprivation of IL-2. GILZ has anti-inflammatory properties and may be involved in signalling modulating apoptosis. Herein we demonstrate that wildtype *Yersinia enterocolitica* which carry the pYV plasmid upregulated GILZ mRNA levels and protein expression in epithelial cells. Infection of HeLa cells with different *Yersinia* mutant strains revealed that the protease activity of YopT, which cleaves the membrane-bound form of Rho GTPases was sufficient to induce GILZ expression. Similarly, *Clostridium difficile* toxin B, another bacterial inhibitor of Rho GTPases induced GILZ expression. YopT and toxin B both increased transcriptional activity of the GILZ promoter in HeLa cells. GILZ expression could not be linked to the inactivation of an individual Rho GTPase by these toxins. However, forced expression of RhoA and RhoB decreased basal *GILZ* promoter activity. Furthermore, MAPK activation proved necessary for profound GILZ induction by toxin B. Promoter studies and gel shift analyses defined binding of upstream stimulatory factor (USF) 1 and 2 to a canonical c-Myc binding site (E-box) in the *GILZ* promoter as a crucial step of its trans-activation. In addition we could show that USF-1 and USF-2 are essential for basal as well as toxin B induced GILZ expression. These findings define a novel way of *GILZ* promoter trans-activation mediated by bacterial toxins and differentiate it from those mediated by dexamethasone or deprivation of IL-2.

## Introduction

*Yersinia enterocolitica* is an enteropathogenic bacterium which causes gastrointestinal disorders such as enteritis and enterocolitis, and extraintestinal manifestations such as lymphadenitis, reactive arthritis, erythema nodosum, uveitis and septicaemia [1], [2]. Host cells can sense *Y. enterocolitica* by recognizing bacterial factors like LPS, invasin, YadA and YopB and can react with a pro-inflammatory response [3], [4], [5].

In line with this, gene expression analysis of epithelial cells revealed that upon interaction with *Yersinia*, chromosomally encoded bacterial factors such as the adhesin invasin lead to gene expression of a large number of pro-inflammatory genes [6]. In cells infected with wildtype *Yersinia* this host response is suppressed by injection of virulence plasmid (pYV)-encoded factors into host cells [7]. In contrast, only a few host genes were found exclusively induced by pYV-encoded factors such as glucocorticoid induced leucine zipper (GILZ) and krueppel like factor (KLF) 2 [6], [8].

GILZ or TSC22 domain family, member 3 (Tsc22d3), a member of the leucine zipper protein family, was identified by comparing mRNA species expressed in dexamethasone (DEX) treated and untreated murine thymocytes [9]. Furthermore, GILZ gene expression was also found to be induced by interleukin (IL) 10 signaling or by IL-2 deprivation in T-cells and macrophages [10], [11]. GILZ expression protects T cells from apoptosis induced by treatment with anti-CD3 monoclonal antibodies, probably via down-regulation of Fas/FasL expression [9]. Most notably, GILZ has been demonstrated to be an important mediator of the anti-inflammatory und anti-proliferative effects of glucocorticoids [12]. For instance it has been shown that anti-inflammatory effects of DEX in lung epithelial cells are mediated by GILZ [13]. It prevents NF-κB activation by inhibition of NF-κB nuclear translocation and DNA-binding due to a direct protein-protein interaction with the NF-κB subunits. [14]. By direct interaction of GILZ with Ras and Raf, the Ras/Raf/Erk signaling pathway is repressed [15], [16]. In similar the activation of the transcription factor AP-1 can be inhibited by GILZ [17].

Here, we have investigated how *Yersinia* may induce GILZ expression. We identified YopT as the crucial toxin which mediates *Y. enterocolitica* induced GILZ expression. Additionally, we present with *C. difficile* toxin B another bacterial toxin inducing GILZ expression. Moreover, we could demonstrate that *GILZ* promoter trans-activation and GILZ protein expression mediated by the Rho-inactivating toxin B and YopT depends on the binding of USF-1 and USF-2 to a canonical E-box element of the *GILZ* promoter, which represents a novel pathway of GILZ induction.

## Results

### *Yersinia Enterocolitica* Induces GILZ Expression in Epithelial Cells

**Figure 1:**
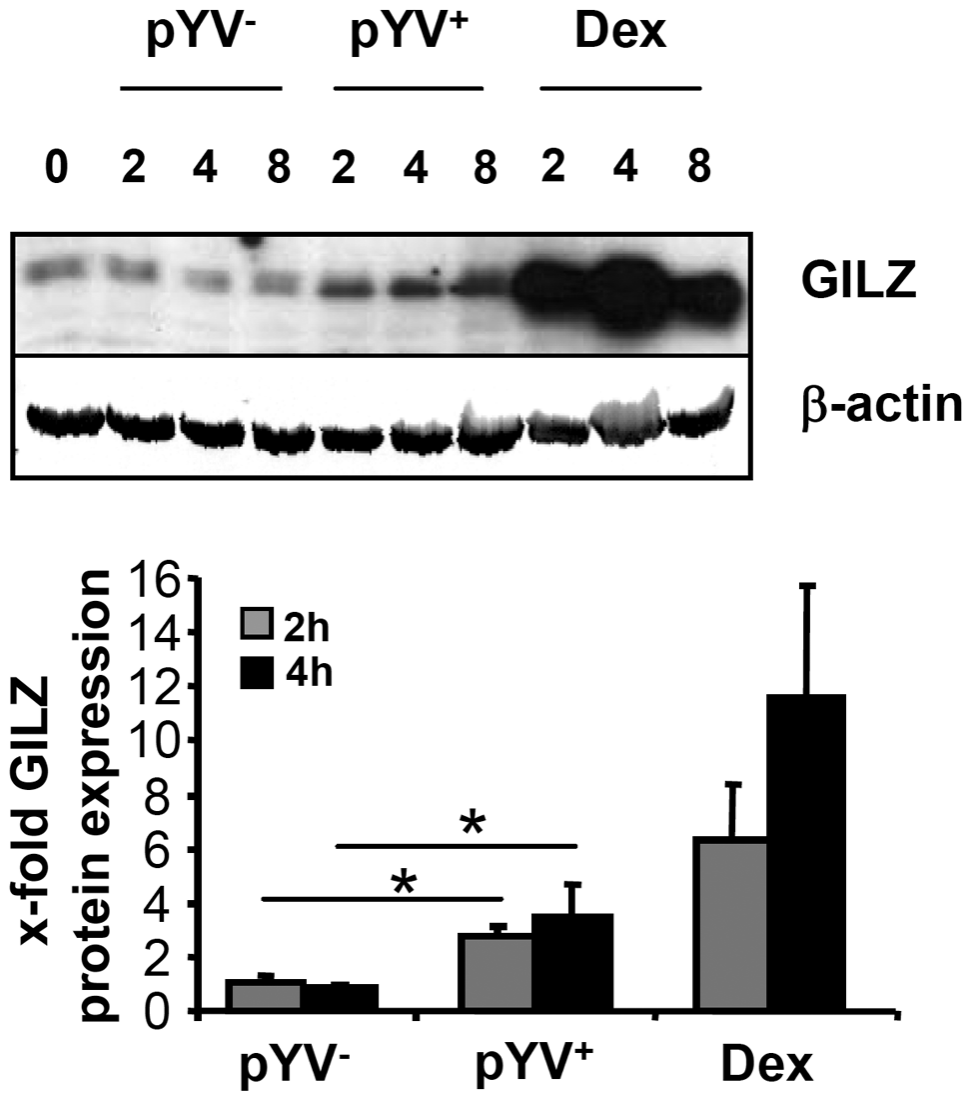
*Yersinia enterocolitica* induces GILZ expression. HeLa cells were infected with a strain harboring the *Yersinia* virulence plasmid (pYV^+^) or plasmid cured strain (pYV^−^) with MOI 20 or stimulated with 100 µM DEX for indicated time intervals. The amount of GILZ in cytosolic proteins lysates of HeLa cells was detected by immunoblot at different time points. Actin was used as an internal standard. A representative experiment and quantification means + SEM normalized to untreated of at least 3 experiments are shown.

Microarray experiments revealed that *GILZ* mRNA was upregulated in epithelial cells upon infection with the *Yersinia enterocolitica* patient isolate WA-314 carrying the pYV virulence plasmid (pYV^+^) [6]. To confirm the previously described microarray experiments, HeLa cells were infected with the p+YV^+^ patient isolate or its virulence plasmid cured derivate pYV^-^. GILZ protein expression was analyzed in cell lysates by Western blotting using a GILZ specific polyclonal antibody preparation at different time points (Figure 1). An induced expression of an approximately 15 kDa immunoreactive band was observed 2, 4 and 8 h after infection with pYV^+^ but not with pYV^-^.

**Figure 2:**
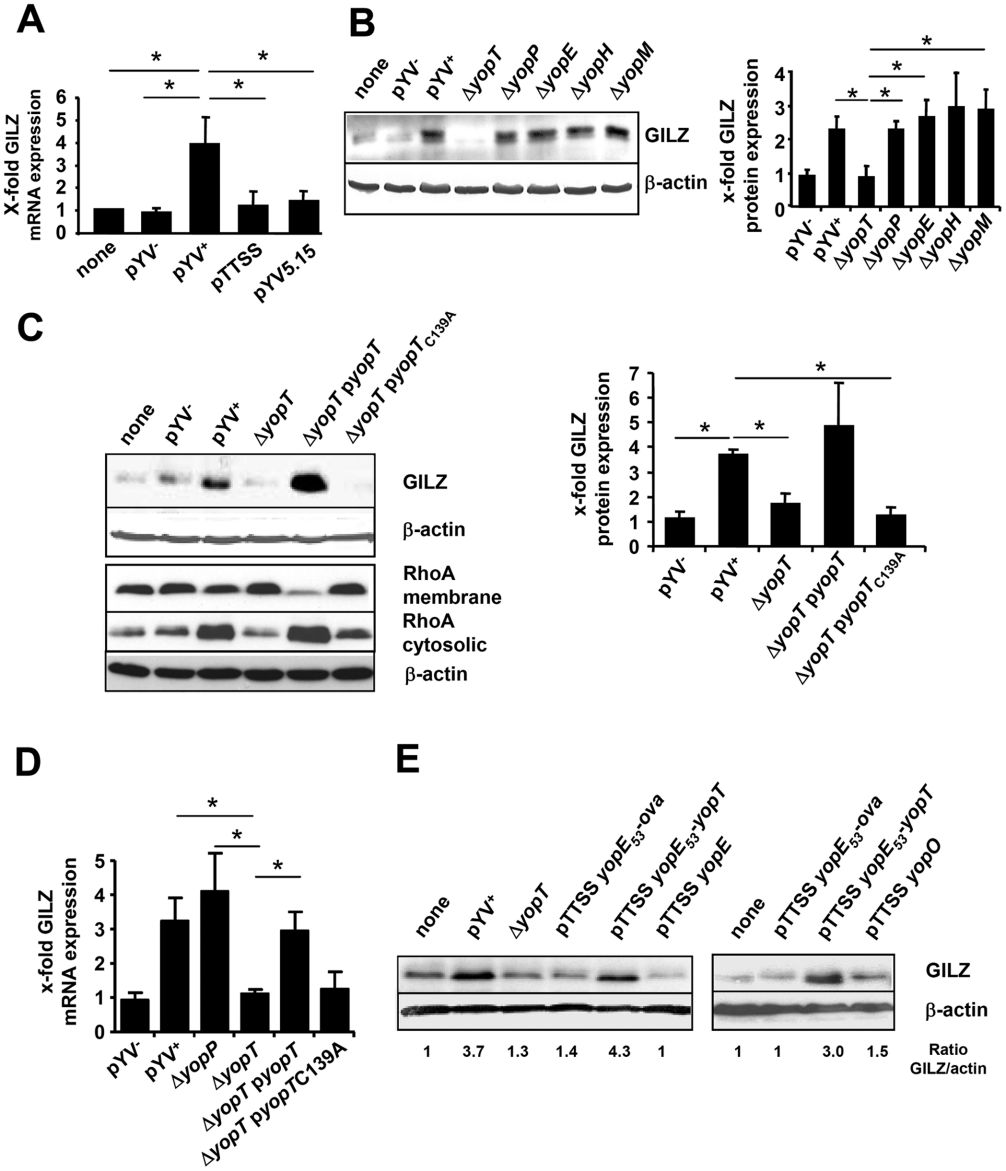
*Yersinia* induced GILZ expression depends on YopT protease activity. HeLa cells were infected with (A) strains with (pYV^+^) or without (pYV^−^) pathogenicity plasmid or with a pathogenicity plasmid derivate coding for a functional translocation apparatus but not for effector Yops (pTTSS) or with a pathogenicity plasmid coding for a defective translocation apparatus (pYV5.15) with MOI 20 for 2 or 4 h to determine *GILZ* mRNA expression by real-time RT-PCR. Means + SD of 2 independent experiments. Significant differences compared to pYV^+^ are indicated by asterisks (p<0.05). (B) Immunoblot of GILZ protein expression induced by strains with single *yop* deletions. Representative immunoblot and quantification means + SEM of 4 independent experiments normalized to untreated. Significant differences compared to ΔyopT are indicated by asterisks (p<0.05). (C) Infection with a *yopT* deletion strain and derivative strains complemented with an additional plasmid encoding wildtype (pyopT) or protease deficient (pyopT_C139A_) YopT. Representative immunoblot analysis of GILZ protein expression in the cytosolic fraction and of RhoA in cytosolic and membrane fraction (left) and quantification means + SD of GILZ expression of two representative experiments normalized to untreated (right). Significant differences compared to pYV^+^ are indicated by asterisks (p<0.05). (D) Experimental design as in C but analysis of GILZ mRNA expression. Significant differences compared to ΔYopT are indicated by asterisks (p<0.05). Mean + SD of 2 independent experiments normalized to untreated. (E) GILZ protein expression induced by strains translocating only one Yop affecting Rho GTPases. Cells were infected with *Yersinia* translocating YopE, YopE_53_-YopT or YopO. Infections with pYV^+^, ΔyopT and a strain translocating only enzymatically inactive YopE_53_-Ova_247–355_ were used as controls.

To investigate whether induction of *GILZ* mRNA expression is dependent on secretion or translocation of *Yersinia* outer proteins (Yops), HeLa cells were infected for 2 h with different *Yersinia* strains such as *Y. enterocolitica* pYV515, a *lcrD* mutant which does not secrete any Yops [18] and *Y. enterocolitica* WA-pTTSS [19] which expresses the type three secretion system and YadA but not YopE, YopP, YopT, YopO, YopM and YopH. *GILZ* mRNA expression was analyzed by real-time RT-PCR. As shown in Figure 2A, only infection with pYV^+^, but not infection with pYV515, pTTSS or pYV^−^ induced *GILZ* mRNA expression, indicating that translocation of effector-Yops is a prerequisite for GILZ protein expression.

To identify those Yops involved in GILZ induction, HeLa cells were infected with different Yop deletion mutants for 4 h and cytosolic proteins were extracted. Western blot analysis revealed that deletion of yopT but none of the other yops tested diminished GILZ protein expression indicating that YopT is involved in the induction of GILZ expression (Figure 2B).

In line with this finding, also infection of the monocyte cell line Mono Mac 6 infection of cells with Yersinia increased GILZ expression in a YopT dependent manner (data not shown). YopT, the most recently identified Yop [20] is a cysteine protease cleaving membrane-bound Rho GTPases from their isoprene membrane anchor [21]. To address whether the protease activity of YopT is essential for induction of GILZ, HeLa cells were infected with a Yersinia pYV^+^ ΔyopT deletion mutant complemented either with a plasmid encoding active YopT (pYV^+^ ΔyopT pyopT) or with a protease-inactive YopT mutant (pYV^+^ ΔyopT pyopT_C139A_) [21] and GILZ protein and mRNA expression was analysed (Figure 2C, D). To ensure that yopT deletion does not affect secretion of other Yops, secretion of YopE and YopH was analyzed but no differences in secretion were found (data not shown). Complementation with yopT restored GILZ expression in contrast to complementation with yopT_C139A_, providing evidence that cysteine protease activity of YopT is crucial for induction of GILZ expression (Figure 2C). In parallel, the effect of YopT on membrane bound and cytosolic RhoA was investigated. Reduction in the amount of membrane bound RhoA was found after infection with pYV^+^ or infection with pYV^+^ ΔyopT pyopT. A clear accumulation of RhoA was found in the cytosolic fraction after infection with pYV^+^ or pYV^+^ ΔyopT pyopT compared to infection with the other strains investigated.

Other virulence factors of Yersinia affect RhoGTPases as well. To investigate whether YopE or YopO also might induce GILZ expression in HeLa cells we used the Yersinia strain pTTSS complemented with plasmids encoding yopE_53_-ova, yopE_53_-yopT, yopE or yopO. Only infection with pTTSS yopE_53_-yopT or pYV^+^ induced GILZ expression (Figure 2E). This data provided no evidence that other bacterial factors beside Yop T contribute to induce GILZ expression.

### YopT Mediated GILZ Induction does not Silence Pro-inflammatory Gene Transcription by NFκB

In response to various inflammatory stimuli, NFκB is released from cytoplasmic sequestration by IκB to the nucleus by ubiquitinylation and subsequent degradation of its binding partner, though it may even play a role in anucleated erythrocytes [22]. In *Yersinia* infection, this activates transcription of pro-inflammatory cytokines, e.g. IL-8 [23], [24]. In line with previous reports showing massively reduced NFκB transcriptional activity in cells over-expressing GILZ [14], we could demonstrate strong inhibition of basal or induced activity of a NFκB promoter luciferase construct in HeLa cells over-expressing GILZ (Figure S1A). Examining the effect of YopT expression on basal and induced NFκB transcription we observed moderate suppression in both cases consistent with results yielded with *Y. pseudotuberculosis* YopT [25]. Furthermore we could link NFκB suppression to YopT protease activity (Figure S1B). However, inhibition of GILZ expression with GILZ specific siRNA (Figure S1C) did not cause significant difference in NFκB transcriptional activity (Figure S1D).

### *Clostridium Difficile* Toxin B Induces Massive GILZ Expression in Epithelial Cells

**Figure 3:**
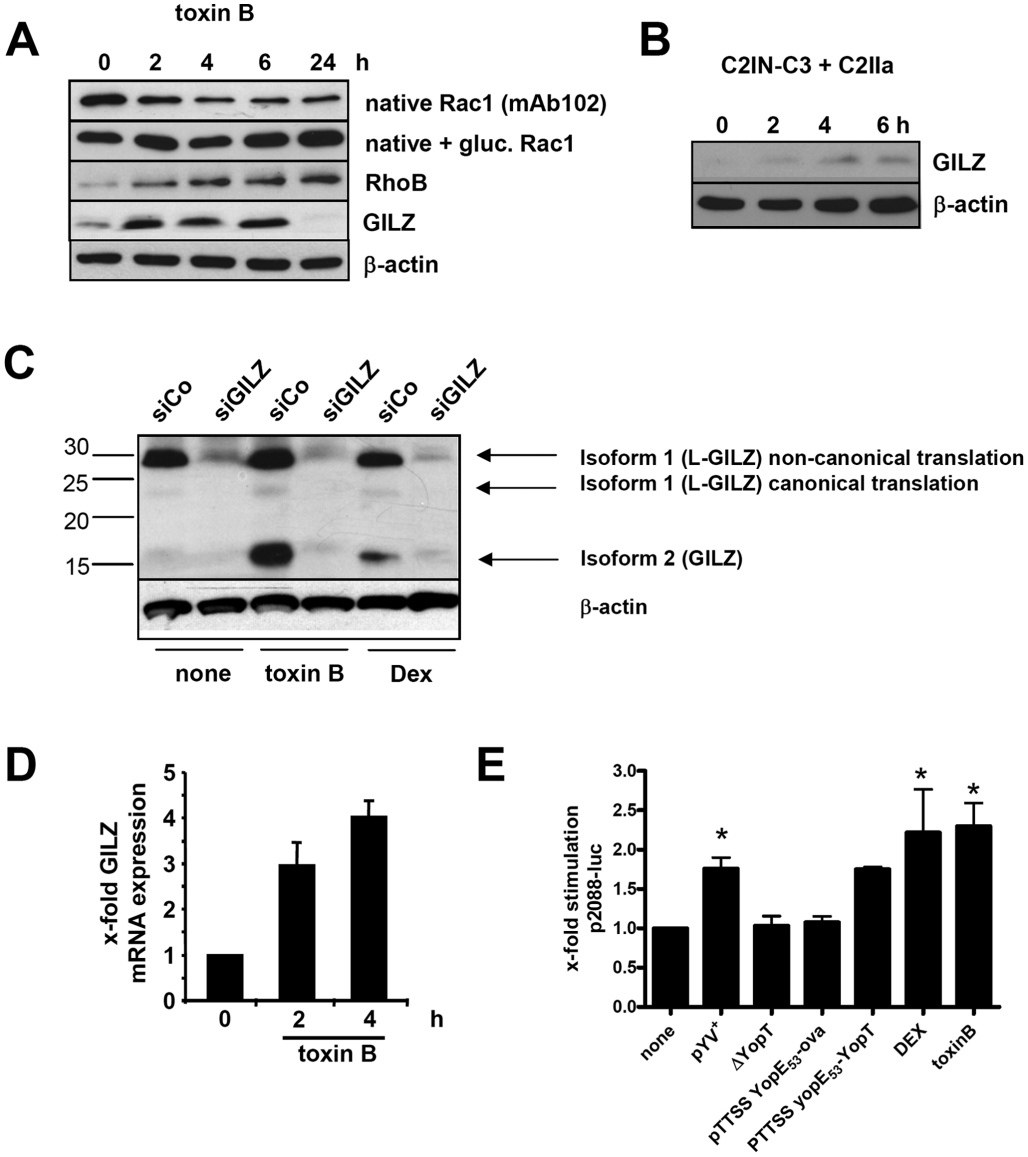
GILZ is expressed upon stimulation with C3 exotoxin or toxin B. (A) HeLa cells were incubated with toxin B (50ng/ml) for indicated the time spans and immunoblots were performed from whole cell lysates using anti-Rac1 mAb102 recognizing only non-glucosylated Rac-1, and an anti-Rac1 mAb antibody recognizing total Rac1 as well as antibodies recognizing RhoB, actin or GILZ. (B) Immunoblot of GILZ and actin expression upon stimulation of HeLa cells with C2IN-C3lim (100 ng/mL) + C2IIa (200 ng/mL) for indicated time spans. (C) To explore the expression of additional GILZ isoforms, HeLa cells were transfected with 7.5 nM siRNA specific for GILZ or control siRNA for 48 h and subsequently either left untreated or stimulated with *C. difficile* toxin B (50 ng/ml) or 100 µM DEX for 4 h. Arrows mark three GILZ isoforms which were inhibited by the used GILZ siRNA. Note that only isoform 1 was induced by the used stimuli. (D) Cells were stimulated with toxin B for 2 or 4 h to determine *GILZ* mRNA expression by real-time RT-PCR. Mean + SD of 2 independent experiments normalized to untreated. (E) To assay transcriptional activity of the GILZ promoter cells were transfected with a luciferase reporter under control of a 2088 bp *GILZ* promoter and co-transfected with pCMV-ß-gal (for standardization) 24 h before infection with a *Y. enterocolitica* pYV^+^ and various mutant strains or treatment with DEX or toxin B. Means + SD of 4 independent experiments normalized to untreated. Significant differences compared to untreated are indicated by asterisks (p<0.05).

The cleavage of the membrane-bound forms of Rho GTPases by YopT leads to their release from membranes, thus abrogating downstream effector contact. We investigated whether other toxins which affect Rho GTPases such as *C. difficile* toxin B (TcdB 10463) or *C. limosum* C3 transferase might also induce GILZ transcription and expression. Toxin B inhibits the small Rho GTPases RhoA, Rac, Cdc42, RhoG and TC10 by glucosylation. The activity of toxin B was indicated by rounding of HeLa cells 2 h after treatment (data not shown). In addition, glucosylation of Rac1 was demonstrated by immunoblots using the antibody mAb102 which only recognizes non-glucosylated Rac1 (Figure 3A). Moreover, toxin B induced RhoB expression as has been previously reported [26] (Figure 3A). C3 transferase exclusively inactivates RhoA, -B and -C by ADP-ribosylation. To enable sufficient uptake, the uptake mechanism of the binary *C. botulinum* C2 toxin was exploited: HeLa cells were treated with a recombinant C2IN-C3lim fusion toxin that is comprised of the catalytic domain of *C. limosum* C3 transferase and the adaptor domain of the C2I component. This allows efficient cellular internalization via the activated C2IIa transport component [27]. The toxic activity of C2IN-C3lim fusion toxin was monitored and validated by induction of typical morphological changes of the cells and by sequential ADP-ribosylation of cell lysates after toxin treatment (Figure S2). GILZ protein expression was analyzed by immunoblots. Toxin B rapidly induced GILZ expression (Figure 3A) while C3 transferase induced GILZ expression only marginally (Figure 3B). In similar, treatment of Mono Mac 6 cells with toxin B or DEX led to increased GILZ expression six hours after stimulation (data not shown).

Recent reports pointed out that several mouse GILZ (Tsc22d3) and probably also human GILZ isoforms exist [28], [29]. So far there are two mouse isoforms annotated in NCBI Protein: Tsc22d3 isoform 1 (NP_001070832.1, 201 aa, 22 kDa) named also long GILZ (L-GILZ) and a shorter isoform 2 (NP_034416, 137 aa, 15 kDa), the prototypic GILZ [28]. Notably, Bruscoli *et al. f*ound L-GILZ to appear at a molecular weight of ∼ 28 kDa. In a recent screening for host factors interacting with herpes simplex protein, human orthologues of these GILZ isoforms have been found: human GILZ isoform 1 (NP_932174.1, 200 aa, 22 kDa) and isoform 2 (NP_004080, 134 aa, 15 kDa) [30]. While the long GILZ isoforms show some differences in the sequence N-terminal of the Tsc22 box, the short isoforms differ only by two amino acids.

Therefore, we addressed the question whether the human orthologue of L-GILZ is also expressed in HeLa cells besides 15 kDa GILZ examined so far. To provide additional evidence, that observed immunoreactive bands really represent GILZ isoforms, cells were transfected with siRNA specific for human GILZ 3′UTR and thus repressing all transcripts of human GILZ. Analysis of HeLa cell lysates showed two dominant bands which were recognized by our polyclonal antibody preparation and which were repressed by the pan-GILZ siRNA: We found a strong and constitutively expressed GILZ protein that migrated at slightly below 30 kDa (human Tsc22d3 isoform 1/L-GILZ) while treatment of cells with DEX or toxin B only markedly induced expression of the 15 kDa GILZ protein (human Tsc22d3 isoform 2; Figure 3C). Our data thus parallel the results of Soundararajan *et al.* who found L-GILZ expressed constitutively in various murine tissues while treatment with the corticoid aldosterone induced GILZ isoform 1 expression [29]. In the presented study we therefore continued focusing on 15 kDa GILZ expression that was also induced by YopT protease activity.

GILZ mRNA expression was analyzed using primers that bound to the GILZ 3-UTR throughout the study. With these pan-GILZ primers also GILZ mRNA expression was also detectable already 2 h after toxin B treatment (Figure 3D).

### Elevated *GILZ* mRNA Levels Result from Transcriptional Activation of the *GILZ* Promoter

To elucidate, whether increased *GILZ* mRNA levels were the result of enhanced transcription, HeLa cells were transfected with a luciferase reporter gene preceded by 2088 bases of the *GILZ* promoter region (p2088-Luc), as such constructs have proven sufficient to assay *GILZ* transcription in previous studies [11]. Measurements of luciferase activity in lysates of cells transfected with p2088-luc were in line with detection of *GILZ* mRNA and *GILZ* protein levels. Significantly increased *GILZ* promoter activity was detected only in cells infected with pYV^+^ or pTTSS yopE53-yopT or treated with toxin B or DEX (as compared to uninfected and untreated cells, Figure 3E. Thus, *GILZ* induction under these conditions results at least partially from enhanced GILZ mRNA de novo synthesis. Notably, toxin B showed comparable potency to activate the GILZ promoter as glucocorticoids.

### Involvement of Rho GTPase – MAP Kinase Signaling in GILZ Induction

**Figure 4:**
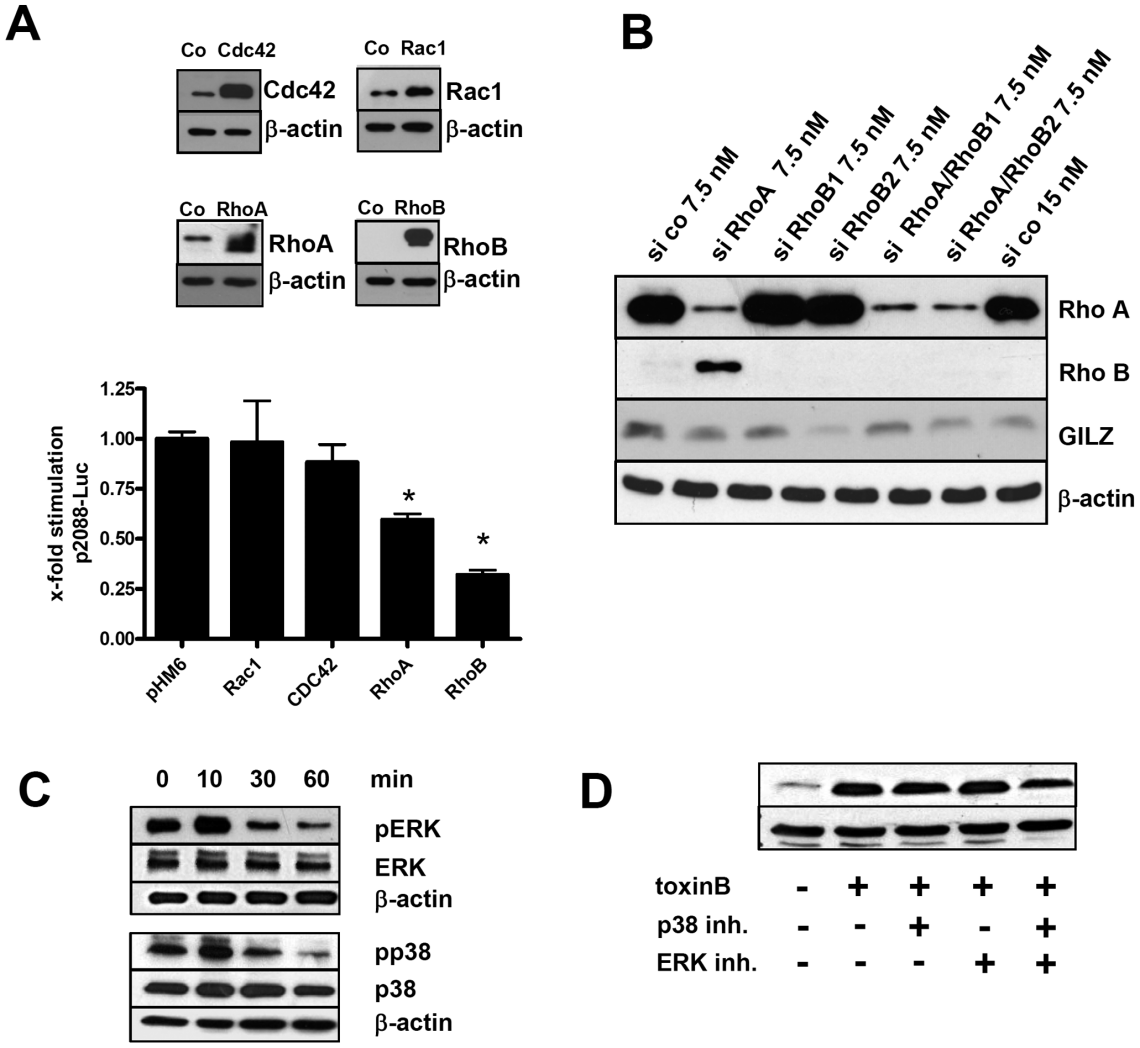
Role of Rho GTPases and MAP kinases for GILZ expression. (A) Overexpression of RhoA or RhoB lowers basal GILZ levels. HeLa cells were co-transfected with the p2088 *GILZ* promoter luciferase reporter and pHM6 based plasmid for overexpression of the indicated Rho GTPases. Means + SD of 3 independent experiments normalized to untreated. Significant differences compared to control vector transfection are indicated by asterisks (p<0.05). In a control experiment, HeLa cells were transfected in the same setting and cell lysates were used for immunoblots to detect RhoA, RhoB, Cdc42 and Rac1 expression. (B) HeLa cells were transfected for 48 h with indicated concentrations of siRNA. Immunoblots were performed from cell lysates for RhoA and RhoB and from cytosolic extracts for GILZ. (C) Toxin B treatment leads to fast and transient MAPK phosphorylation. After treatment of HeLa cells with toxin B for the indicated time spans, levels of phosphorylated as well as total ERK and p38 were assayed by immunoblot. (C) Toxin B induced GILZ expression is mediated by both ERK and p38 MAPK. Cells were pretreated with MAPK phosphorylation inhibitors SB 202190 (p38) or PD 98059 (ERK) 2 h prior to toxin B stimulation and GILZ protein was detected by immunoblot analysis at 6 h or 24 h after stimulation.

Further studies of GILZ induction were focused mainly on toxin B because of its stronger trans-activation of the *GILZ* promoter. The observation of GILZ induction by YopT and toxin B but not by YopE or YopO and only marginally by C3 toxin indicated that inactivation of Rho family GTPases may not be solely sufficient for potent GILZ induction. Concordantly, inhibition of the effector protein Rho associated kinase kinase (ROCK), an important transducer of RhoA signaling as well as treatment of cells with lovastatin, a general isoprenylation blocker of Rho family GTPases were largely ineffective in generating GILZ expression (data not shown). To investigate whether modulation of Rho GTPase expression levels may affect the transcriptional activity of the GILZ promoter, first HeLa cells were transfected with different expression vectors leading to overexpression of RhoA, Rac1, CDC42 or RhoB. Transient transfection led to overexpression of these proteins compared to transfection with empty control vector (Figure 4A, upper panel). Interestingly, overexpression of RhoA or RhoB specifically and significantly repressed basal *GILZ* promoter activity, as opposed to expression of Rac1 or CDC42 (Figure 4A, lower panel). This result clearly indicates that distinct Rho GTPases modulate transcriptional regulation of GILZ. In a second experiment HeLa cells were transfected for 48 h with either siRNA specific for RhoA or RhoB or both and immunoblots were performed (Figure 4B).RhoB expression was hardly detectable but inhibition of RhoA expression led to upregulation of RhoB expression. Neither inhibition of RhoA alone nor of RhoA and RhoB in combination led to upregulation of GILZ expression, indicating that expression levels of RhoA and RhoB might be involved in modulating transcriptional activity but are not sufficient for regulation of GILZ expression.

As toxin B has been reported to elicit ERK and p38 phosphorylation in treated cells [31], [32] we examined the possible contribution of MAPK activation in toxin B mediated GILZ induction. We detected moderate p38 and ERK phosphorylation peaking as early as 10 min after toxin B treatment which subsequently vanished (Figure 4C).

To test whether MAPK activation contributed to GILZ induction, we analyzed the ability of toxin B to induce GILZ expression in the presence of inhibitors of MAPK phosphorylation and treated cells with SB 202190 (target: p38) or PD 98059 which neutralizes ERK phosphorylation. While single treatment with p38 or ERK phosphorylation inhibitors did not affect GILZ induction, treatment with both inhibitors in combination reduced GILZ expression, to some extent 6 h after toxin B treatment (Figure 4D).

In conclusion, induction of GILZ expression by bacterial toxins did not only require the inhibition of Rho GTPases. We speculate that several activities of these toxins are necessary, such as both inhibition of distinct RhoGTPases and activation of MAP kinases.

### Toxin B Transactivates the GILZ Promoter via a Canonical E-box

**Figure 5:**
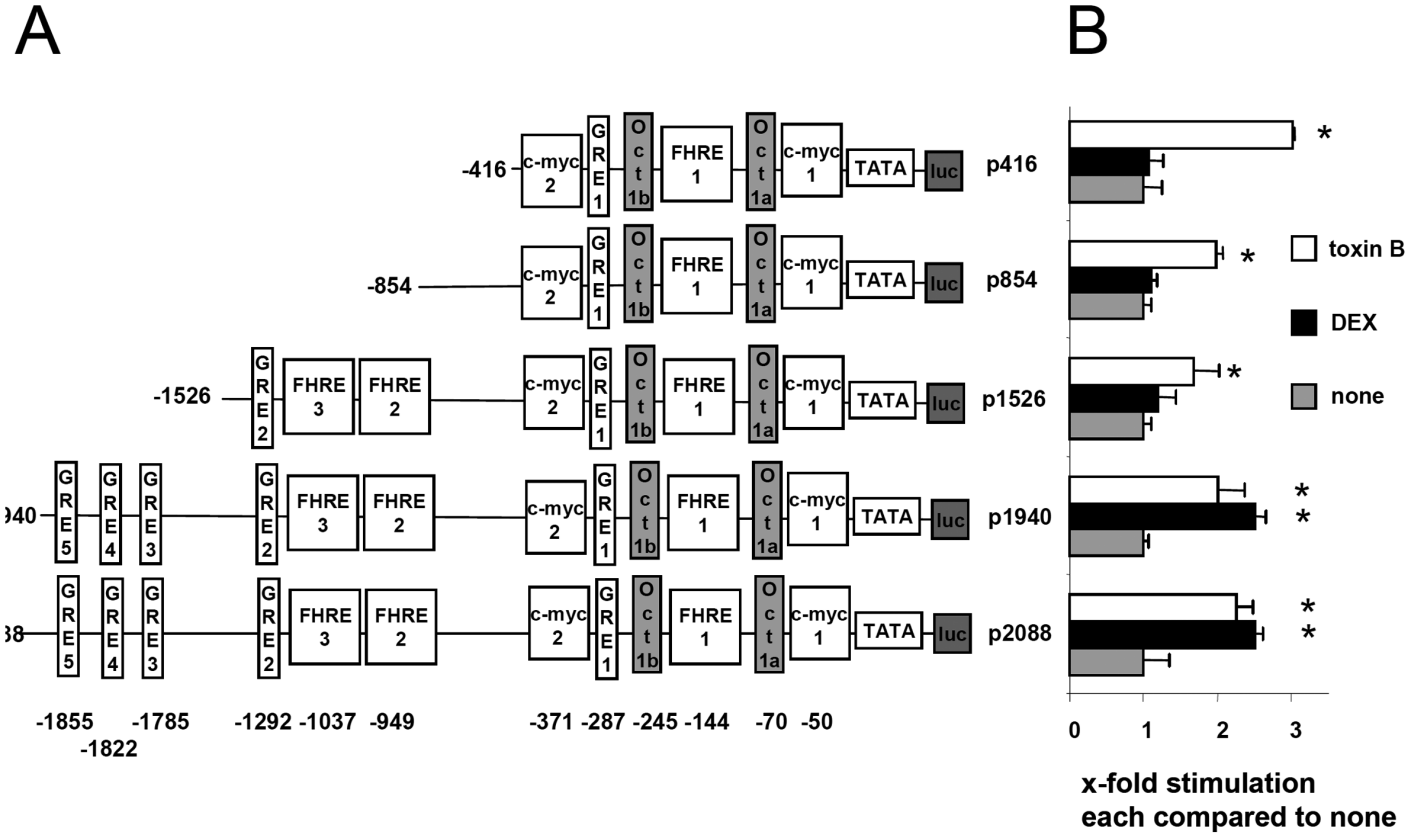
Differential activation of truncated *GILZ* promoter elements by toxin B or DEX. HeLa cells were transiently transfected with pCMV-ß-gal for 24 h and co-transfected (A) with p2088-Luc or luciferase reporters fused to shortened promoter regions, containing the indicated TF binding sites. GRE: glucocorticoid responsive element, FHRE: forkhead responsive elements, c-myc: c-myc binding site (E-box), Oct: octamer binding site (B) Transfected cells were treated with 100 µM DEX or 50 ng/ml toxin B for 6 h. Subsequently luciferase assays were performed. Results are expressed as fold induction compared to unstimulated (none) cells and represent the mean + SEM of three experiments performed in triplicates (p<0.05).

To test which promoter elements might be of relevance for toxin B induced GILZ expression HeLa cells were transfected with *GILZ* promoter-luciferase constructs containing promoter fragments of different length (ranging from −416 bp to −2088 bp upstream of the transcription initiation site, Figure 5A). As reported previously [11], [33] removal of the glucocorticoid responsive elements (GRE) 3, 4 and 5 (−1526 construct) abrogated DEX mediated promoter activation. In the case of toxin B stimulation however, transfection with even the most truncated *GILZ*-promoter fragment (−416) proved sufficient for trans-activation (Figure 5B).

**Figure 6:**
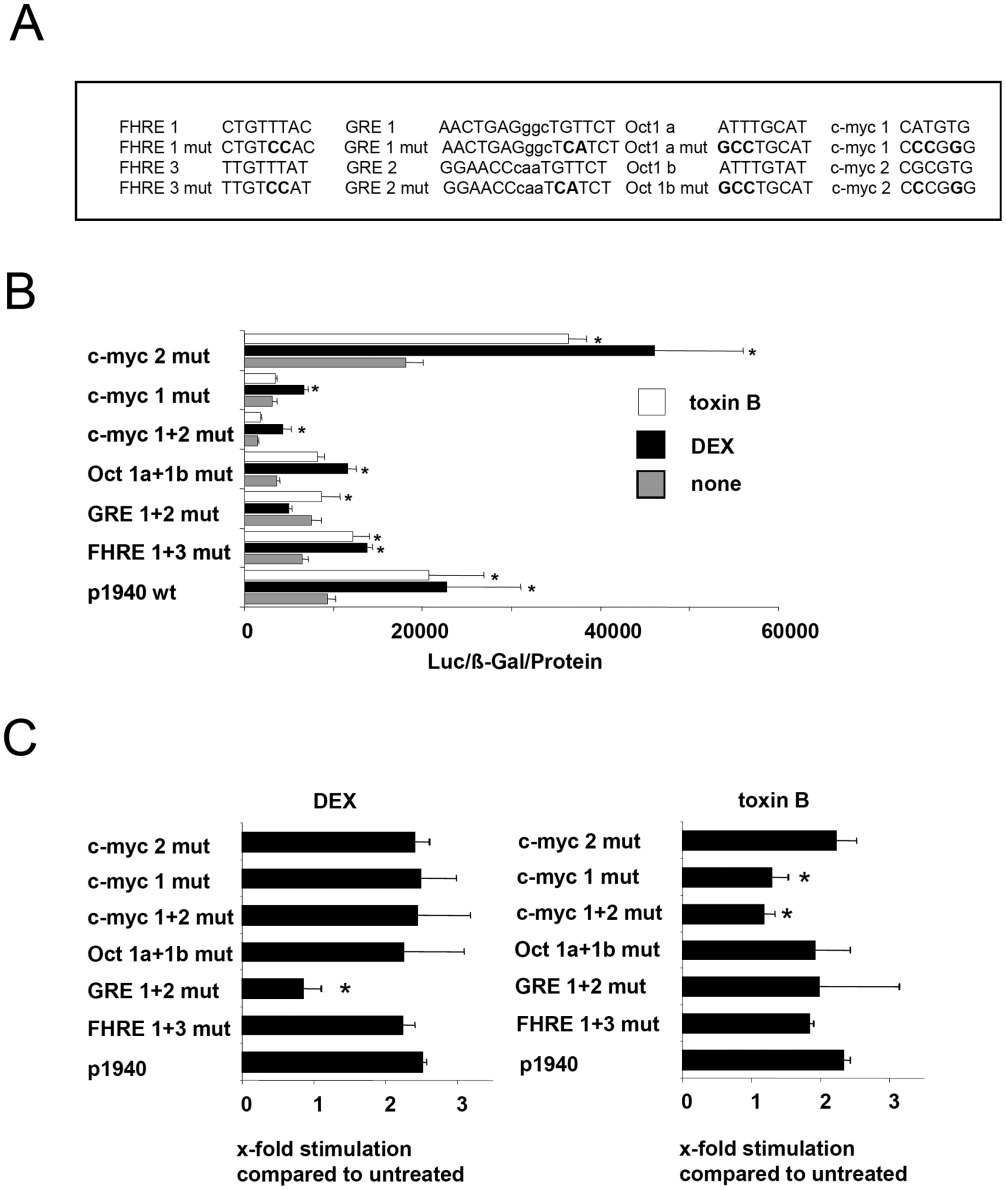
Importance of specific cis-elements for toxin B induced GILZ promoter trans-activation. (A) Recognition sequences of cis-elements which were mutated. Mutated base pairs are highlighted using bold letters. (B) HeLa cells were transfected with p1940-Luc and different mutated derivatives for 24 h and subsequently stimulated with DEX or toxin B for 6 h. Data are shown as relative light units (RLU) standardized to β-Gal activity and protein concentration or (C) as fold induction after DEX or toxin B stimulation compared to untreated conditions of each individual expression vector. Means + SEM of four independent experiments are shown. Asterisks indicate significant differences between DEX or toxin B stimulation compared to uninfected (p<0.05).

Conserved transcription factor (TF) binding sites in this sequence comprise a FHRE (FHRE1), two Oct1 (Oct1a and Oct1b) and one GRE element (GRE1), a canonical E-box (myc1) and a non-canonical E-box (myc2). To single out the promoter motif required for *GILZ* induction by toxin B we introduced into the *GILZ*-1940 promoter region point mutations disrupting consensus sequences of identified trans-activating sites (Figure 6A). Measurement of luciferase activity in HeLa cells transfected with this derivates of the p1940 *GILZ* promoter luciferase construct revealed decreased luciferase activity of all mutants except for myc2 compared to p1940 (Figure 6B). This data indicates that FHRE1, Oct1, GRE elements and the canonical E-box myc1 positively affect basal activity of the *GILZ* promoter while the non canonical E-box myc2 enables repression of basal *GILZ* promoter activity. While FHRE1, Oct1 and myc2 mutated promoters retained their responsiveness to toxin B- and DEX-treatment, relative increase of *GILZ* promoter activity (compared to unstimulated cells transfected with the same construct) by DEX was only influenced by mutation of GRE1+2. In contrast, *GILZ* promoter trans-activation induced by toxin B was only affected by mutation of the canonical E-box myc1 (Figure 6C).

### Toxin B Treatment and pYV^+^ Infection Results in TF Binding to the *GILZ* Promoter Canonical E-box

**Figure 7:**
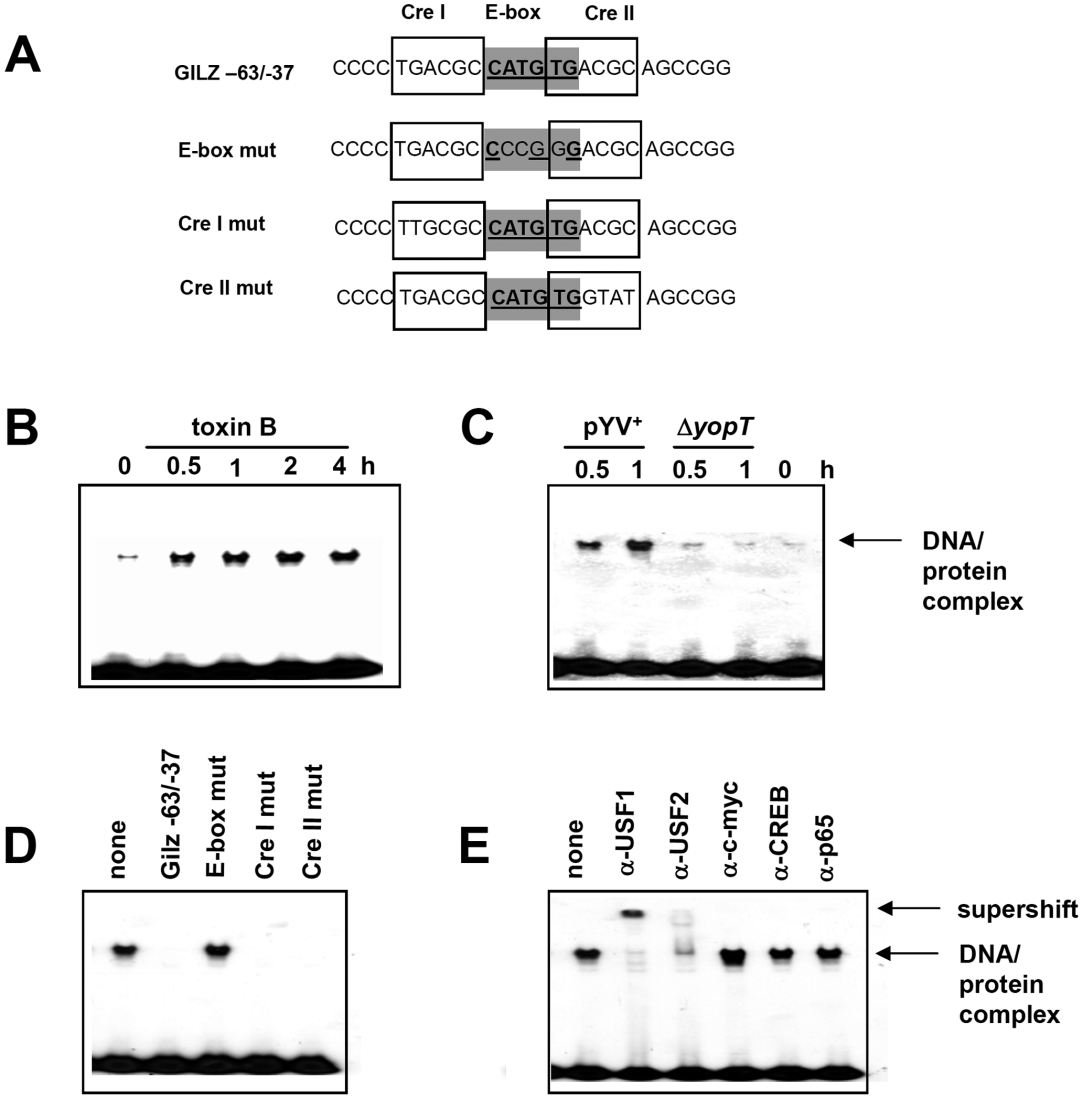
Role of myc-1 E-box in TF binding. Electromobility shift analyses were performed using a double-stranded oligonucleotide probe representing the *GILZ* promoter sequence −63 to −37 and for some experiments probes with mutations of the E-box cis-elements and the flanking cAMP response (Cre) elements as depicted in (A). HeLa cells were stimulated/infected for 0.5 h or indicated time intervals with toxin B (B, D, E) or *Y. enterocolitica* (C) and nuclear extracts of these cells were incubated with P^32^-labeled GILZ−63/−37 probe. Subsequently band shift analyses were performed. (D) Nuclear extracts were pretreated with a 100-fold excess of indicated cold probes. (E) Nuclear extracts were pretreated with indicated antibodies. Anti-p65 antibody was used as a negative control.

To identify proteins which may bind to the canonical E-box (myc1), electrophoretic mobility shift assays (EMSA) were performed using a *GILZ* promoter dsDNA fragment (*GILZ* -63/−37) comprising the canonical E-box and two partially overlapping putative Creb binding elements (CRE) (Figure 7A). Stimulation of HeLa cells with toxin B as well as infection with *Y. enterocolitica* pYV^+^ - but not Δ*yopT* - increased protein complex binding to *GILZ* −63/−37 as soon as 30 minutes after stimulation with toxin B (Figure 7B and C). Competition experiments revealed that binding of TF protein could be abrogated by addition of excess unlabeled *GILZ*−63/−37 DNA probe, CRE I mut or CRE II mut probe (Figure 7D). In contrast, an excess of cold probe with mutated myc1 E-box (E-box mut) was not able to compete with *GILZ*−63/−37. We therefore conclude that the E-box but not the flanking CRE elements are crucial for increased binding of transcription factors to this *GILZ* DNA region after treatment of cells with toxin B.

### Toxin B Treatment Results in *GILZ* Promoter Activation by USF1 and USF2

The CANNTG E-box sequence has been characterized as binding motif of the large basic/helix-loop-helix/leucin zipper (b/HLH/Z) TF family [34]. The human c-Myc oncoprotein is probably the most prominent member of this family, but co-incubation with antibody directed against c-Myc did not change gel-shift behavior of the *GILZ*−63/−37 probe as was the case with antibodies specific for Creb and p65 that have been included as negative controls (Figure 7E).

In HeLa cells, the b/HLH/Z TF upstream stimulatory factor (USF) has been described to bind the E-box motif with high affinity and to activate transcription of downstream genes [35].

Addition of anti-USF-1 or anti-USF-2 antibody to EMSA samples specifically abrogated or supershifted DNA-protein complex formation, respectively (Figure 7E). This data shows that toxin B induced *GILZ* promoter activation is associated with increased binding of USF-1 and USF-2 to the canonical E-box.

**Figure 8:**
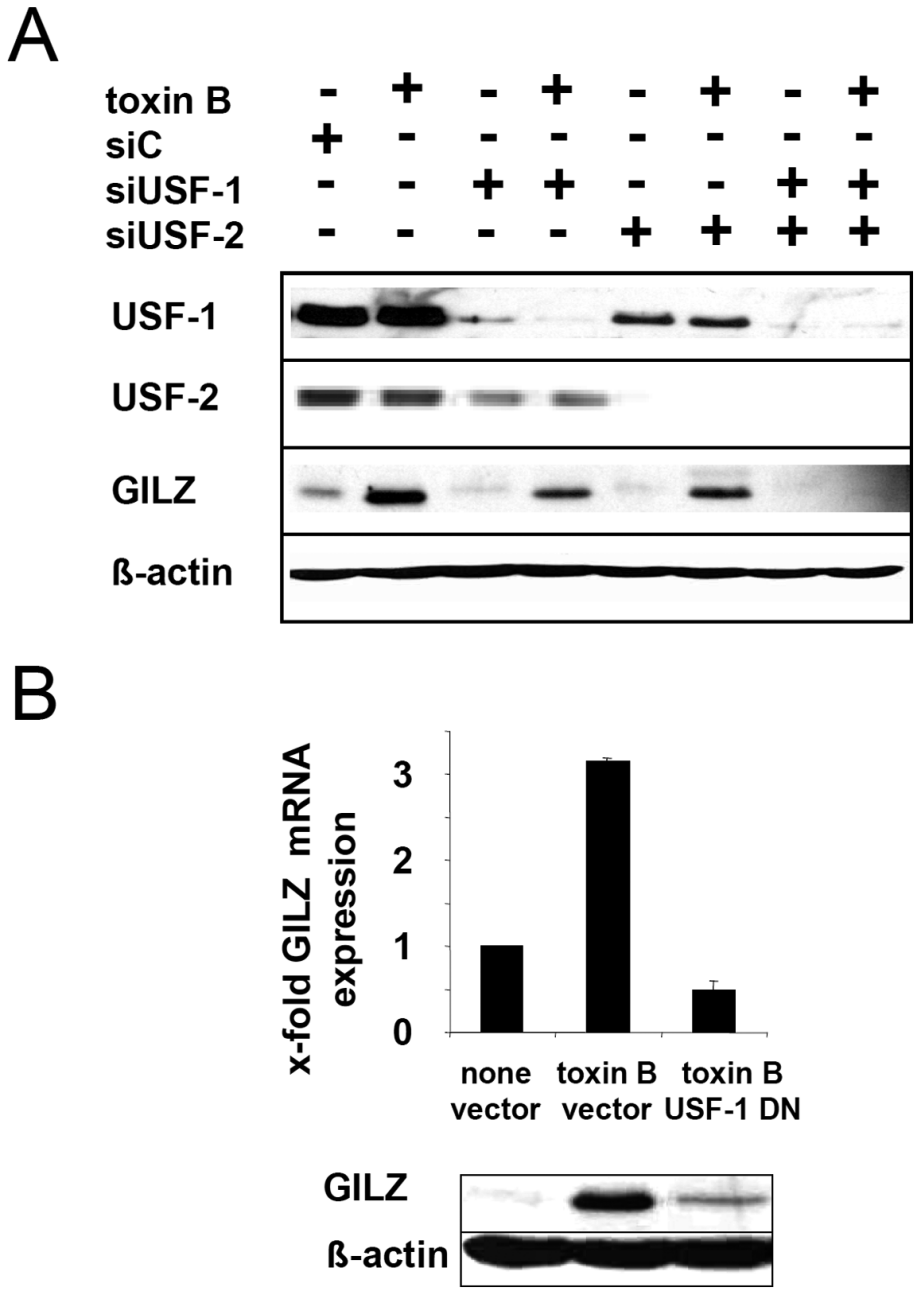
Role of USF-1 and USF-2 for toxin B induced GILZ expression. (A) HeLa cells were transfected with siRNA silencing USF-1 or USF-2 or with control siRNA (siC) 48 h prior to toxin B treatment and USF-1/2 as wells as GILZ and actin expression was determined by immunoblot. (B) HeLa cells were transfected with empty vector or a dominant negative (DN) USF-1 mutant and subsequently *GILZ* mRNA or GILZ protein expression was determined by real time RT-PCR (one representative experiment performed in quadruplicates, means + SEM) or immunoblot.

Inhibition of both USF-1 and USF-2 protein expression by transfection with USF-1 and USF-2 siRNA abrogated basal as well as toxin B-induced GILZ protein expression (Figure 8A). In line with this finding, transfection with an expression vector encoding dominant negative USF-1 reduced toxin B induced GILZ protein expression effectively (Figure 8B).

### GILZ Induction does not Modulate Toxin B Induced Apoptosis

Looking for other effects that GILZ expression induced by bacterial toxins might exert, we looked on induction of apoptosis by large clostridial toxins like toxin B in HeLa cells and other epithelial cell lines [36], [37]. GILZ had been shown to mediate pro-apoptotic as well as anti-apoptotic signaling [9], [12], so we examined if GILZ might be a mediator or modulator of toxin B induced apoptosis. GILZ induction by toxin B was silenced effectively by GILZ specific siRNA (Figure S3A). HeLa cell apoptosis induced by toxin B was measured by Nicoletti assay after 24 h or 48 h. Pre-treatment with GILZ specific or control siRNA however, made no significant difference in the proportions of apoptotic cells after toxin B treatment (Figure S3B). Thus, GILZ induction by toxin B did not modulate the extent of its ability to induce apoptosis.

## Discussion

Upon interaction with epithelial cells, *Y. enterocolitica* pYV^+^ inhibits gene expression induced by other bacterial factors such as Inv, YadA and YopB very efficiently [3], [4], [5], [6]. Only a few genes are upregulated in epithelial cells infected with *Y. enterocolitica* pYV^+^ [6]. As demonstrated here, infection of HeLa cells with *Y. enterocolitica* results in activation of the *GILZ* promoter followed by enhanced gene transcription and GILZ protein expression.

Previous studies showed that several GILZ isoforms exist in mice as well as in humans [28], [29], [30]. Research has been focused on the 15 kDa isoform 2 so far, but it has become clear that other isoforms may be differentially expressed and may display different downstream activities. Thus, these studies also analyzed the expression of the larger isoform 1 (L-GILZ). In various murine tissues and cultured kidney epithelial cells, L-GILZ was found to be constitutively expressed, while GILZ expression was induced by aldosterone treatment [29]. In primary mouse myoblast cultures however, DEX treatment induced both L-GILZ and GILZ expression [28].

We transfected HeLa cells with siRNA matching the 3′UTR of GILZ to prove that detected protein bands represent indeed GILZ protein. By using polyclonal GILZ antibody, we detected strong constitutive expression of L-GILZ at almost 30 kDa. In fact, the L-GILZ transcript encodes only a 22 kDa protein, when translated from the canonical translation start codon. However, it has been demonstrated in mouse cells that translation of this GILZ isoform starts earlier at a non-canonical CUG codon, thus resulting in a larger protein [28]. Comparison of mouse (NM_001077364.1) and human (NM_198057.2) GILZ mRNA transcripts revealed that the same non-canonical start codon preceded by a Kozak consensus sequence is also present in human GILZ mRNA and our western blot analysis showed that human L-GILZ is also predominantly translated as a larger protein in HeLa cells, while a faint band indicates that low amonts of the canonical translation product also exist.

Additional stimulation or infection of HeLa cells only induced expression of 15 kDa GILZ isoform 2, further referred to as GILZ in this study. Unfortunately, there is no uniform nomenclature of GILZ isoforms. We have adopted the numbering of GILZ isoforms that is used by NCBI Protein, but the nomenclature of other databases (e.g. UniProt) or publications may be completely different.

Infection with different *Yersinia* mutants which are not able to secrete or translocate Yops demonstrates that translocation of Yops into the cytosol HeLa cells is necessary for GILZ induction. In addition we provide evidence that YopT but not YopE or YopO which are also known to inhibit Rho GTPases is required to induce GILZ expression.

Infection of HeLa cells with Yop deletion mutants revealed that GILZ expression was abrogated upon infection with a *Y. enterocolitica yopT* deletion mutant and complementation with *yopT* restored GILZ expression. In contrast, deletion of *yopP*, *yopE*, *yopM* or *yopH* did not affect GILZ expression, implying that YopT might be crucial for GILZ expression.

Infection with a mutant translocating only YopT into HeLa cells clearly induced GILZ expression indicating that no other Yops are required to induce GILZ expression. Consistently mutants translocating YopE or YopO were not able to induce GILZ expression.

For catalytic protease activity of YopT the amino acids C139, H258 and D274 are crucial [38]. In line with this finding infection experiments with a *yopT* deletion mutant complemented with *yopT*_C139A_ encoding protease deficient YopT demonstrated that protease activity of YopT was required for increased GILZ expression.

YopT is a cysteine protease which cleaves RhoA, Rac, and Cdc42 [21], [38], [39] directly upstream of the C-terminal isoprenylated cysteine. Which Rho GTPases are affected by YopT inside the cells is still unclear. It has been reported that translocated YopT inserts into the plasma membrane. There it cleaves RhoA but not Rac or Cdc42 removing membrane bound RhoA as well as depleting the cytoplasmic RhoA - GDI-1 pool. This finally results in accumulation of RhoAn the cytosol [40]. Other publications however indicate that upon *Yersinia pseudotuberculosis* infection YopT cleaves RhoA as well as Rac and Cdc42 from nascent phagosomes and affects RhoG [41], [42].

Since YopT is able to induce GILZ expression, we tested whether other bacterial toxins inhibiting Rho GTPases could also trigger GILZ expression. In line with this hypothesis *C. difficile* toxin B which leads to UDP-glucosylation of Rho GTPases [43] and inactivates RhoA, Rac, Cdc42, RhoG and TC10 or indirectly activates RhoB [44], strongly induced GILZ expression two, four and six h after start of treatment. After 24 h GILZ expression faded, possibly because of toxin B degradation and because of the activation of RhoB expression that in turn may inhibit GILZ expression. In contrast, C3 transferase which affects RhoA, -B, and -C by ADP-ribosylation only marginally induced GILZ expression. The low impact of C3 transferase on GILZ expression might suggest that inactivation of RhoA, RhoB or RhoC is not sufficient to trigger significant *GILZ* expression. Similarly, YopE does not induce GILZ expression. YopE possesses GTPase activating activity that has been shown to reduce RhoA, Rac1, and Cdc42 activity *in vitro* [45] but seems to act preferentially on Rac1 and RhoG *in vivo* [42], [46]. Evidence that modulation of Rho GTPases may affect GILZ expression was provided by data showing that overexpression of RhoA or RhoB partially inhibited transcriptional activity of the *GILZ* promoter. However, inhibition of either RhoA, RhoB or both using siRNA did not upregulate GILZ expression. On the one hand these data argue that inhibition of those Rho GTPases may play a minor or no role for GILZ expression. On the other hand they also emphasize that we are far away from understanding all the consequences of enzymatic modifications of GTPases induced by pathogenic bacteria.

Searching for additional stimuli, we focused on the strong toxin B induced GILZ expression. Besides inhibiting Rho GTPases, toxin B is also activating pro-inflammatory signaling. Earlier reports [31], [32], [47] pointed out that treatment of cells with toxin B resulted in MAP kinase activation that is independent of Rho GTPase UDP-glucosylation. Since inhibition of MAP kinases inhibited GILZ expression partially, our results suggest at least a minor contribution of p38 as well as ERK activity in enhancing toxin B induced GILZ expression.

Interestingly, the kinetic of MAPK phosphorylation was much faster and much more transient than that found by Na *et al.* after toxin B treatment of the NCM460 colonocyte line, where ERK phosphorylation peaked at 2 h post stimulation and lasted for hours [32]. Therefore we have no evidence for a mechanism of autocrine TGF-*a* signaling demonstrated to cause ERK phosphorylation by their report. However, the same kinetic of p38 and ERK phosphorylation was found to trigger maturation of toxin A-stimulated dendritic cells [31], suggesting that besides TGF-*a* signaling there might be a second mechanism of early MAPK activation by toxin B in some cell types.

IL-10 is a known inducer of GILZ expression [10], [48]. Therefore, autocrine or paracrine IL-10 signaling induced by bacterial toxins [49] could be a mediator of GILZ induction. However, it is very unlikely that IL-10 acts as an effector for indirect toxin B induced GILZ expression, because pre-treatment of toxin B stimulated HeLa cells with 100 to 200 ng/ml neutralizing anti-human IL-10 antibody (JES3-29F1, BD Pharmingen) had no impact on toxin B induced GILZ expression (data not shown).

Taken together, rather than suggesting a linear induction pathway, expression of (inducible isoform 2) GILZ appears to be regulated by diverse signals induced by some pathogenicity factors.

To shed some light on the downstream events of GILZ induction by YopT and toxin B we examined activation of the GILZ promoter. Previous studies of GILZ promoter activation revealed two principal mechanisms so far: IL-2 deprivation leads to binding of FoxO3 to the forkhead responsive elements (FHRE) and transcriptional activation. Deletion of the three distal glucocorticoid responsive elements of the GILZ promoter abrogates DEX mediated transcriptional activation of GILZ, demonstrating a crucial role of GRE elements for DEX mediated GILZ expression [11], [33]. Our data show that similar to DEX, toxins modifying GTPases such as *C. difficile* toxin B and YopT lead to trans-activation of the GILZ promoter. Further studies using shorter GILZ promoter elements demonstrated that in contrast to DEX, a truncated promoter fragment starting at −416 is sufficient for toxin B-induced GILZ promoter trans-activation, however.

Mutations affecting *cis*-elements in this region revealed that only mutation of a canonical E-box (myc1) abrogates toxin B induced *GILZ* promoter activity. This further differentiates *GILZ* induction by toxin B from that promoted by IL-2 deprivation or glucocorticoids and defines a novel activation mechanism of the *GILZ* promoter. In line with this finding the binding of transcription factors to this canonical E-box increased after stimulation with toxin B or infection with *Y. enterocolitica* pYV^+^ but not with a Δ*yopT* deletion mutant. This strongly supports the idea that toxin B and YopT use a common downstream pathway to induce GILZ expression.

Previous studies reported interaction of the ubiquitously expressed upstream stimulatory factors (USF)-1 and -2 with canonical E-box regulatory elements (CANNTG) [34]. Concordantly, binding of USF-1 and -2 to the *GILZ* E-box CATGTG could be demonstrated after toxin B treatment. Knock-down of USF-1 and USF-2 with siRNA or out-competition of USF-1 by a dominant negative mutant clearly demonstrated USF-1 and USF-2 as key elements in toxin B-mediated *GILZ* expression. To our knowledge this is the first report showing *GILZ* trans-activation by USF and also the first piece of evidence on USF-meditated gene expression in response to bacterial virulence factor action.

In addition, we tested the hypothesis whether YopT induced GILZ expression may contribute to *Yersinia* mediated inhibition of NF-κB activity. By overexpression of GILZ we could demonstrate that GILZ is able to inhibit NF-κB activation in epithelial cells. Our data are in line with an earlier report showing that GILZ inhibits NF-κB activation in T cells [14]. Having shown that YopT induces GILZ and that GILZ inhibits NF-κB activation, we postulated that YopT might also be involved in *Yersinia*-mediated inhibition of NF-κB activation. However, while infection with *Y. enterocolitica* pTTSS-*yop*E_53_-*yop*T translocating only YopT into HeLa cells led to GILZ expression, inhibition of NF-κB activity was not achieved (data not shown). It has been published earlier that *Y. pseudotuberculosis* YopT moderately inhibited NFκB activation [25]. We could reproduce this finding, however in our hands only overexpression of YopT into HeLa cells resulted in some inhibition of NF-κB activity. Moreover an association between YopT induced GILZ expression and the slight reduction of NF-κB activity could not be shown. While the difference in the effectiveness of YopT translocated while *Yersinia* infection is most likely due to the use of different strains, the pathways of YopT mediated NFκB inhibition in either case are probably the same. As discussed by Viboud *et al*. [25] Rho GTPases promote IκB degradation by JNK activation [50] or by the PAK- ERK pathway [51]. Removal of Rho GTPases from their membrane anchor by YopT therefore should inhibit these ways of NFκB activation and account for the decreased NFκB activity resulting from YopT action.

We also investigated a possible role of GILZ in the induction of apoptosis by toxin B which has been shown to trigger apoptosis by caspase dependent as well as independent pathways. However, knock-down of GILZ did not significantly change the ratio of apoptotic cells.

Therefore, the physiological relevance of GILZ in bacterial infections stays elusive. Several studies highlighted putative physiological effects of GILZ in mice which might potentially modulate immune responses against *Yersinia*. On the one hand, transgenic mice overexpressing GILZ show augmentation of thymocyte apoptosis [52], a bias to Th2 development [53] and inhibition of NF-κB signaling and Th1 response, leading to protection of mice in a colitis model [54]. On the other hand LPS, an important component triggering inflammatory response in bacterial infection dampened GILZ expression in cultured alveolar macrophages as well as in lung tissues [55] indicating that bacterial factors not only upregulate but also downregulate GILZ expression. The respective contributions of the opposing effects of different bacterial components thus may influence the magnitude and the role of GILZ expression in bacterial infections. For instance, in case of *C. difficile* infection it was shown that the *C. difficile* toxin A and the here characterized GILZ inducer toxin B induce a strong inflammatory response [56] and are responsible for the acute inflammation in the gut characterized by pseudomembrane formation [57]. However, besides the toxins A and B several other components of *C. difficile* like surface layer proteins [55] or flagella also induce inflammation [56]. Interestingly the pro-inflammatory response and intestinal secretion in the gut induced by C. difficile toxin A can be reduced by exogenous DEX treatment and is also down-regulated by endogenous glucocorticoid production [58]. From this data, one may speculate that both toxin B as well as glucorticoid induced GILZ might be of physiological relevance during *C. difficile* infection in limiting the pro-inflammatory response and intestinal secretion. The study of GILZ knockout may help to verify this hypothesis.

*GILZ* knockout mice lacking all four isoforms were recently described and it was demonstrated that GILZ expression results in infertility of male mice but had no impact on inflammatory responses at all [59]. Thus for instance, no difference in the pro-inflammatory response after simultaneous treatment with DEX and LPS was observed between bone marrow derived macrophages of *GILZ* knockout or wildtype control mice indicating that GILZ has rather no impact on inflammatory responses which is in line with our data. Nevertheless, these mice might prove useful to elucidate the role of GILZ in mouse models of *Yersinia enterocolitica* or *Clostridium difficile* infection.

Taken together we could clearly demonstrate that - in addition to previously shown trigger mechanisms - some bacterial toxins such as YopT and toxin B induce GILZ expression. In contrast to dexamethasone treatment and IL-2 deprivation, toxin B and *Yersinia* YopT mediated regulation of GILZ expression depends on binding of USF-1 and USF-2 to the *GILZ* promoter. These results open a new branch of investigation in the field of host pathogen interactions.

## Materials and Methods

### Bacterial Strains and Growth Conditions

All bacteria were grown in Luria-Bertani broth (LB). For infection with *Y. enterocolitica* strains or *E. coli* HB101 pInv1914 (*E. coli* pInv1914) [60], overnight cultures grown at 27°C or 37°C, respectively, were diluted to an OD_600_ of 0.2 in LB and incubated for 3 h at 37°C. All bacterial strains used in this study are listed in Table 1. The strains Δ*yopT pyopT*, or Δ*yopT pyopT*_C139A_ were generated by electroporation of the plasmids pIM279 or pISO1, respectively, into the strain ΔyopT [61].

**Table 1:** Bacterial strains used in this study.

Designation	Genotype or Description	Reference or source
pYV^+^	*Y. enterocolitica* serotype O:8; clinical isolate;WA-314 pYVO8^+^	[68]
pYV^-^	Serotype O:8, virulence plasmid cured derivative of*Y. enterocolitica* WA-314 (WA-C)	[68]
WA-314 pKD46	WA-314 pKD46	[69]
Δ*yopE*	WA-C pYV Δ*yopE* 17–203	[69]
Δ*yopH*	WA-C pYV Δ*yopH*17–455	[69]
Δ*yopM*	WA-C pYVΔ*yopM* 17–351	[69]
Δ*yopP*	WA-C pYVΔ*yopP* 17–272	[69]
Δ*yopT*	WA-C pYVΔ*yopT*	[61]
pYV^+^Δ*yopT* p*yopT*	WA-C harbouring pYVΔ*yopT* and pBC18R P_lac_ *yopT*, *sycT* (pIM279)	[20], [61], this study
Δ*yopT* p*yopT*_C139A_	WA-C harbouring pYVΔyopT and pBC18R P_lac_ *yopT*_ C139A_, *sycT* (pISO1)	[61], [70], this study
pYV505	Tn5 insertional inactivation of *lcrD*	[18]
pTTSS	WA-C with plasmid pTTSS encoding the type three secretionsystem and YadA but lacking YopE, YopH, YopM, YopO, YopTand YopP	[71]
pTTSS *yopE*_53-_ *ova*	WA-C pTTSS with plasmid pBME53-Ova_247–355_	[72]
pTTSS *yopE*_53_-*yopT*	WA-C pTTSS with plasmid pBME53-yopT	[72]
pTTSS *yopE*	WAC pTTSS with plasmid pBM-yopE	[72]
pTTSS *yopE*_53_-*yopP*	WAC pTTSS with plasmid pBM53-yopP	[72]
E.coli pInv 1914	E. coli HB101 strain expressing *Y. enterocolitica* Inv	[66]
pCJE138-G1	*Hind*III - *Sal*I fragment of *yopE* 138-*gfp* in pACYC184; *Hind*III –*yopE*138 - *Bam*H1 - *gfp* - *Sal*I	[73]
pTTSS *yopO*	WA-C pTTSS with plasmid pBM-*yopO*	[74], this study

### Generation of Yersinia Mutants

To generate the pTTSS *yopO* strain secreting only YopO but no other Yops, the plasmid pBM-*yopO* was electroporated into WA-314 pTTSS and positive colonies were screened by selection with chloramphenicol. To yield the plasmid pBM-*yopO* the pYV plasmid of wild type *Y. enterocolitica* strain WA-314 was used as a template to amplify the *yopO* gene in two fragments, thereby eliminating the internal *Bam*HI site using the primers 5′-GGA TCC ATG AAA ATC ATG GGA ACT ATG TCA CC-3′ and 5′-GTG TCT GGT CCA GAT GCT TCT GAA TCC TCT GCA GTG AAG GTT CGA CG-3′ for fragment 1 and 5′-GTC GAC TCA CAT CCA TTC CCG-3′ and 5′-ATC GTC GAA CCT TCA CTG CAG AGG ATT CAG AAG CAT CTG GAC CAG ACA CAC-3′ for fragment 2. Both fragments were used as templates in a PCR yielding the unified yopO gene, using the primers 5′-GGA TCC ATG AAA ATC ATG GGA ACT ATG TCA CC-3′ and 5′-GTC GAC TCA CAT CCA TTC CCG-3′. The *sycO* gene together with the *sycO* and *yopO* promoter region was amplified using the primers 5′-AAG CTT GAC TGT GCG CCG ACA CG-3′ and 5′-GGA TCC GCT TTA CTC ATC CCC ATT TAA TAA C-3′. The PCR fragments of *yopO* and *sycO* were subcloned into pCR2.1 TA (Invitrogen, Karlsruhe, Germany) and sequenced (Medigenomix, Martinsried, Germany). The fragment containing *sycO* and the promoter region was inserted between the *Hind*III and *Bam*HI sites of pACYC184 thus completing the generation of pBM-*yopO*.

### DNA Constructs

The *GILZ* promoter (p2088) comprising the nucleotide sequence from –2088 to +20 flanked by *Kpn*I and *Hind*III sites was inserted into pGL3 basic (Promega, Madison, WI) resulting in p2088-Luc [11]. The generation of the constructs p1940, p1526, p854, p416, GRE 1+2 mut and FHRE 1+3 mut has been described elsewhere [11], [33]. Point mutations were introduced into the plasmid p1940-Luc by the Quick change PCR based strategy (Stratagene, Cedar Creek, USA) to generate the additional constructs using the following phosphorylated primers: c-myc 1-mut, 5′-GAC GCA GCC GGC TCC TCC-3′, 5′-CCG GGG CGT CAG GGG CCA TGC-3; c-myc 2 mut, 5′GTC CAG GGA GTA TGA CAT GGG AG-3′, 5′-CCG GGC CCT CAC CAT CAC G-3′, Oct-1a, 5′-TGC ATG CCC CTG ACG CTG-3′, 5′-GGC GAG TCC TGT ACC GGG CTT TGT GG-3′; Oct-1b 5′ TGT ATT TCT TAT TTC TCT AGA AAT CAG CTC CAG-3′. To generate the double mutants Oct-1a+1b mut and c-myc 1+2 mut, the vectors c-myc 2 mut and Oct1a were used as templates.

In order to generate the expression plasmid pHM6-*GILZ*-HA, the coding sequence of human *GILZ* was amplified from cDNA of dexamethasone stimulated HeLa cells using the primers 5′-GCA GCC AAG CTT GAT GAA CAC CGA AAT GTA TCA G-3′ (fwd) and 5′-GGA CAC GTG CAC TTA CAC CGC AGA ACC-3′ (rev) and ligated into pCRII Blunt TOPO (Invitrogen). The *Hind*III/*Pml*I *GILZ*-fragment of pCRII Blunt-*GLZ* was then ligated into the expression vector pHM6 (Roche Diagnostics Mannheim, Germany).

The plasmids pHM6-YopT or pHM6-YopT_C139A_ were generated by PCR using the primers 5′GGT ACC ATG GAC AGT ATT CAC GGA CAC-3′ (fwd) and 5′-GCG GCC GCC GTT AAA CCT CCT TGG-3′ (rev) and as templates pIM279 or pISO1, respectively. After ligation into pCRII Blunt TOPO, the amplicons were cut with *Kpn*I/*Not*I and ligated into pHM6 resulting in pHM6-YopT and pHM6-YopT_C139A_.

For constitutive expression of Rho GTPases in mammalian cells we generated the following constructs: RhoA: pHM6-RhoA_F25N_-HA was yielded by amplification of *RhoA*_F25N_ inserted into pGEX-2T [62] using the primers 5′-ACT CAT AAG CTT GGC TGC CAT CCG GAA GAA ACT GGT G-3′ (fwd) and 5′-ACA GTA GAA TTC ACA AGA CAA GGC AAC CAG-3′ (rev). After ligation into pCRII Blunt TOPO, *RhoA*_F25N_ was donated as a *Hind*III/*Eco*RI fragment to pHM6. Cdc42/RhoB/TC10: Coding sequences contained in pRK5 [63] have been amplified with 5′-GGT ACC ATG CAG ACA ATT AAG TGT GTT GT-3′ (fwd) and 5′-GCG GCC GCT CAT AGC AGC ACA CAC CT-3′ (rev) (*Cdc42*), 5′-AAG CTT GAT GGC GGC CAT CCG C-3′ (fwd) and 5′-GAA TTC TCA TAG CAC CTT GCA GCA GTT GAT GCA G-3′ (rev) (*RhoB*) or 5′-AAG CTT GAT GCC CGG AGC CGG CCG C-3′ (fwd) and 5′-GCG GCC GCT CAC GTA ATT AAA CAA CAG3’ (rev) (*TC10*). After ligation into pCR2.1 TOPO, inserts were donated as *Kpn*I/*Not*I (*Cdc42*), *Hind*III/*Eco*RI (*RhoB*) or *Hind*III/*Not*I (*TC10*) fragments to pHM6. Rac1: pCDNA3.3-HA-Rac1 was a kind gift from Klaudia Giehl.

All PCR amplificates have been checked by sequencing prior to subcloning. pNFκB-Luc was purchased from Stratagene (Amsterdam, Netherlands) and pCMV-β-galactosidase (pCMV-ß-gal) from Clontech (Palo Alto, CA), pUHD-USF1mutBR (USF-1 DN) was a kind gift from M. Eilers [64].

### Cell Culture and Infection

HeLa cervical epithelial cells (ATCC CCL-2.1) were grown in RPMI 1640 (Biochrom KG, Berlin, Germany) supplemented with 10% fetal bovine serum (Sigma Chemical, St. Louis, MO), 2 mM L-glutamine (Biochrom KG; Berlin, Germany), penicillin (100 U/ml), and streptomycin (100 µg/ml) (Biochrom KG; Berlin,Germany) in a humidified 5% CO_2_ atmosphere at 37°C.

For infection, cells were washed with PBS and incubated in antibiotic-free medium. Cells were infected with a MOI of 20 or as indicated and incubated at 37°C. After 1 h gentamicin (100 µg/ml) was added to kill extracellular bacteria. At indicated time points after infection, cells were lysed to perform subsequent analyses.

### Transfection of siRNA

siRNA was purchased from Qiagen, Hilden, Germany and comprised the following sequences (sense strand): siUSF1, 5′-GAC CCA ACC AGU GUG GCU AdTdT, siUSF2, UAG CCA CAC UGG UUG GGU CdTdT-3′ [65], siC 5′-UUC UCC GAA CGU GUC ACG UdTdT-3′ (AllStars negative control siRNA), siGILZ 5′-CAC CCU GUU GAA GAC CCU G dTdT-3′. SiRNA transfection was done using HiPerfect (Qiagen;Hilden, Germany) transfection reagent according to the manufactureŕs fast-forward protocol, adding first siRNA mixture and then cells (6·10^4^ HeLa cells in the 24-well format for luciferase experiments, 5·10^5^ HeLa cells in the 6-well format for western blot analysis). Minimal siRNA concentrations yielding extensive knockdown were applied to avoid off-target effects. End concentrations were 100 nM for siUSF1/2 and 10 nM for siGILZ. SiRNA transfection was performed 72 h prior to further treatment when using siUSF-1/2, or 48 h for GILZ siRNA.

### Transient DNA Transfection

For DNA transfection, 5·10^4^ HeLa cells were seeded in 24-well plates and transfected on the following day in Opti-MEM (Invitrogen; Karlsruhe, Germany) with DNA constructs using ExGen transfection reagent (Fermentas, Ontario, Canada) according to the manufacturer’s instructions. After 4 h, the transfection medium was replaced by the normal culture medium and cells were incubated for another day at 37°C. For luciferase assays cells were transfected with 125 ng of the respective luciferase reporter construct and co-transfected with 125 ng of pCMV-ß-gal to assay transfection efficiency. For expression of Rho GTPases, GILZ, USF-1 DN or YopT, cells were transfected with 250 ng of the respective expression plasmids.

### Stimulants/Inhibitors

*Clostridium difficile* toxin TcdB10463 was kindly provided by Dr. I. Just (Institute of Toxicology, Hannover Medical School, Hannover, Germany). For delivery of C3 transferase into cells, the C2IN-C3lim fusion toxin was used together with the activated transport component C2IIa from the *C. botulinum* C2 toxin [27]. Dexamethasone was purchased from Sigma Chemical (St. Louis, MO).

MAPK inhibitors SB 202190 and PD 98059 were purchased from Calbiochem (E. Merck, Darmstadt, Germany). Both inhibitors were used at final concentrations of 10 µM. Inhibitor treatment started 1 h before toxin B treatment and was continued until cells lysis. Cell viability was confirmed by microscopic observation.

### Quantitative RT- PCR Analysis

Total RNA of infected HeLa cells in 6-well plates was extracted using the RNeasy Mini Kit (Qiagen, Hilden, Germany). 2 µg of RNA were reverse transcribed as described earlier [66]. Real-time PCR was performed on a LightCycler (Roche Diagnostics, Mannheim, Germany), using LC-FastStart DNA Master SYBR Green Kit (Roche Diagnostics; Mannheim, Germany). *GILZ* mRNA expression was quantified using the primers 5′-GGG CAG AGC CAC TAA ACT TG-3′ and 5′-GCC TTC ACG AAA CAG AGG AG-3′. As a standard an amplicon generated with these primers was used. The Real-time PCR was carried out with an annealing temperature of 68-58°C (−0.5°C/s) for 45 cycles. *GILZ* mRNA expression levels were normalized to *glucose-6-phosphate-dehydrogenase* RNA expression levels, detected by Real-time PCR using primers and DNA standard of Search LC as described by the manufacturer (Search LC, Heidelberg, Germany). Results were analysed using the LightCycler software.

### Antibodies and Immunoblotting

Antibodies used for EMSAs and immunoblotting were purchased from Serotec, Düsseldorf, Germany (rabbit anti-CREB (KL2)), from Santa Cruz Biotechnology, Santa Cruz, CA (rabbit anti-human Myc (N-262), rabbit anti-human USF-1 (H86), rabbit anti-human USF-2 (C20), mouse anti-RhoA (26C)), from Cytoskeleton Inc, Chicago, USA (mouse anti-Rac1 mAb, mouse anti-CDC42 mAb), from BD Bioscience, Heidelberg, Germany (mouse anti-Rac1 clone 102) and from Cell Signaling Technology, Boston, MA (rabbit anti-RhoB). Polyclonal antibodies directed against GILZ were prepared by immunizing rabbits (Biogenes, Berlin, Germany) with a GST-GILZ fusion protein. Subsequently, serum was affinity purified using recombinant GST-GILZ protein. To detect GILZ 4 h or as indicated after infection/stimulation by immunoblotting, cells were lysed by three freeze/thaw cycles in PBS containing complete protease inhibitor cocktail (Roche Diagnostics, Mannheim, Germany) plus DTT (400 µM). Cell fragments were spun down by centrifugation at 15,000 g for 10 min at 4°C. In assays were the membrane fraction was analysed the pellet was dissolved in a buffer containing 150 mM NaCl, 20 mM Tris-HCL, pH 7.6, 1% Triton X and protease inhibitor cocktail. Protein concentration was determined by the Bradford method using Bio-Rad protein assay (Bio-Rad, Hercules, CA) and up to 120 µg of protein were loaded on a 15% SDS gel. Separated proteins were transferred electrophoretically to an Immobilon-P PVDF membrane (Millipore, Bedford, MA). The membrane was blocked with 50% soy milk. For detection of GILZ expression the polyclonal rabbit anti-GILZ antibody was used followed by incubation with a goat anti-rabbit IgG (H+L) as a secondary antibody and a horseradish peroxidase-conjugated (HRP) donkey anti-goat IgG (H+L) antibody (Jackson Immuno Research Laboratories, West Grove, PA) as a tertiary antibody. Immunoblots were stripped and actin expression was determined by using a mouse anti-actin antibody (Sigma-Aldrich, St. Louis, MO) and HRP labeled rabbit anti-mouse IgG antibody (DAKO, Glostrup, Denmark). For determination of USF-1 and USF-2 expression as secondary antibody a HRP labeled goat-anti rabbit IgG antibody (Santa Cruz Biotechnology, Santa Cruz, CA) was used. The detection was carried out using the chemiluminescence detection kit (ECL) from Amersham Biosciences (Uppsala, Sweden).

### Luciferase Reporter Assay

To evaluate luciferase activity, the Luciferase Reporter Gene Assay, high sensitivity (Roche Diagnostics, Mannheim, Germany) was used. Cells were washed twice with PBS and lysed with luc lysis buffer according to the manufacturer’s instructions. Lysates were centrifuged and supernatants removed for determination of protein content, β-galactosidase and luciferase activity. Luciferase activity was standardized on ß-galactosidase activity and protein concentration (relative light units).

### Sequential ADP-ribosylation of Rho Proteins

The ADP-ribosylation status of Rho was determined by sequential ADP-ribosylation. To this end, the cell lysates were incubated for 20 min at 37°C with biotin-labelled NAD^+^ and C2IN-C3lim (100 ng/mL). The proteins were separated by SDS-PAGE, blotted onto nitrocellulose and the biotin-labelled, i.e. ADP-ribosylated Rho was detected with streptavidin-peroxidase by Western blotting.

### Electrophoretic Mobility Shift Assays (EMSA)

EMSAs were performed as described by [24] using the double stranded probe (sense strand) GILZ –63/−37 5′-CCC CTG ACG CCA TGT GAC GCA GCC GG-3′. For competition experiments a 100-fold excess of unlabeled probes, namely GILZ –63/−37, E-box mut 5′-CCC CTG ACG CCC CGG GAC GCA GCC GG-3′, CRE I mut ´5′-CCC CTT GCG CCA TGT GAC GCA GCC GG-3′ and CRE II mut 5′-CCC CTG ACG CCA TGT GGT ATA GCC GG-3′ were included in the binding reaction. For supershift analyses, different antibodies were included in the binding reaction. Samples were resolved on a 5% nondenaturating polyacrylamide gel using 0.5 x TBE (25 mM Tris-HCl, 25 mM boric acid, 0.5 mM EDTA) as running buffer. Gels were transferred to Whatman 3M paper and dried under vacuum. Protein binding was assessed via autoradiography.

### Measurement of Apoptosis

Apoptosis was measured as the fraction of cells with hypoploid nuclei [67]. Briefly, cells were harvested 24 h or 48 h after treatment, washed and resuspended in hypotonic lysis buffer containing propidium iodide. After 10 min incubation on ice, one volume of PBS was added and cells were analyted on a FACSCalibur Cytometer (BD Biosciences; Heidelberg, Germany).

### Statistics

If not stated otherwise, data shown in the figures are from representative experiments. Comparable results were obtained if not otherwise stated in a total of at least two experiments. Differences between mean values were analyzed using either one-way ANOVA or the Student’s *t* test. *P*<0.05 was considered statistically significant.

## Supporting Information

Figure S1 **Overexpression of GILZ or YopT inhibits NF-κB promoter activity.** HeLa cells were transfected with pNF-κB-Luc luciferase reporter and pCMV-β-gal for 24 h and (A) co-transfected with either pHM6 or pHM6-GILZ. Subsequently HeLa cells were infected with *E. coli* pInv1914 expressing invasin (MOI 100) for 6 h and luciferase assays were performed. Means + SD of two independent experiments are shown. (B) HeLa cells were transfected with pNF-βB Luc and pCMV-ß-Gal and co-transfected with pHM6, pHM6-YopT or pHM6-YopT_C139A_ for 24 h. Subsequently HeLa cells were left untreated or infected with *E. coli* pInv1914 expressing invasin (MOI 100) for 6 h and luciferase assays were performed. Results of A and B are expressed as fold induction compared to uninfected cells treated with empty vector control and represent the mean + SEM of a representative experiment performed in quadruplicates. Asterisks indicate a significant difference between NF-κB activation of cells transfected with pHM6 compared to cells transfected with pHM6-YopT (p<0.05). (C, D) HeLa cells were transfected with siGILZ and cultured for 24 h and subsequently transfected with pHM6 or pHM6-YopT for additional 24 h. Cell lysates were harvested to determine GILZ and β-actin expression by immunoblot. NF-κB driven luciferase activity was assayed described for B. Means + SEM of three independent experiments.
(TIF)

Figure S2 **HeLa cell intoxication by C3 toxin Rho ADP-ribosylation.** HeLa cells were incubated at 37°C with C2IN-C3lim (100 ng/mL) + C2IIa (200 ng/mL). After 2, 4 and 6 h pictures were taken to demonstrate the C3-induced change in cell morphology (A) and the cells were lysed. B. The percentages of cells showing “C3-morphology” were calculated from the pictures. Values are given as mean ± S.D. (n = 3); ** p<0.005. C. The ADP-ribosylation status of Rho from the cells was determined by sequential ADP-ribosylation. To this end, the cell lysates were incubated for 20 min at 37°C with biotin-labelled NAD^+^ and C2IN-C3lim (100 ng/mL). The proteins were separated by SDS-PAGE, blotted onto nitrocellulose and the biotin-labelled, i.e. ADP-ribosylated Rho was detected with streptavidin-peroxidase by Western blotting. The ADP-ribosylated Rho is shown. Comparable amounts of blotted lysate proteins were confirmed by Ponceau S-staining (not shown). (Note: In this experimental setting unlabeled Rho ADP-ribosylation in the intact cells competes with biotin-labelled ADP-ribosylation after lysis. A strong signal therefore means that Rho was not ADP-ribosylated in the intact cells, a weak signal indicates ADP-ribosylation of Rho by the toxin in the intact cells prior to lysis).
(TIF)

Figure S3 **Impact of GILZ on toxin B triggered apoptosis.** HeLa cells were transfected with siGILZ for 48 h (A) and subsequently stimulated with Toxin B for additional 24 h or 48 h. Apoptotic cells were detected by Nicoletti assay. Results are expressed as mean + SEM of three independent experiments (B).
(TIF)
