# Supplementary figures and images for: Yersinia enterocolitica YopT and Clostridium difficile Toxin B Induce Expression of GILZ in Epithelial Cells
Source: PLoS One. 2012 Jul 9;7(7):e40730. doi: 10.1371/journal.pone.0040730 (PMC3392236; doi:10.1371/journal.pone.0040730)

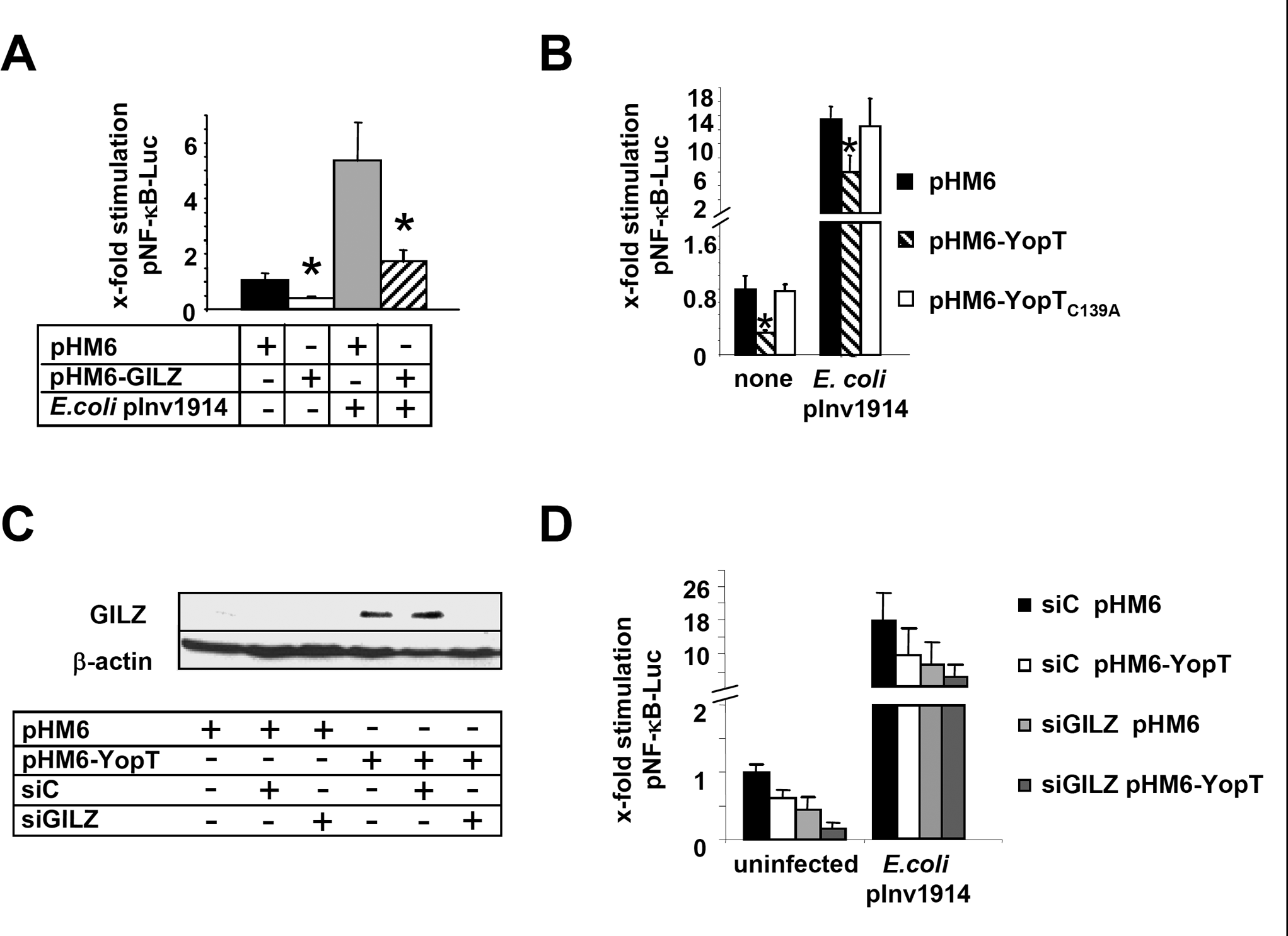

Supplement: Figure S1 — Overexpression of GILZ or YopT inhibits NF-κB promoter activity. HeLa cells were transfected with pNF-κB-Luc luciferase reporter and pCMV-β-gal for 24 h and (A) co-transfected with either pHM6 or pHM6-GILZ. Subsequently HeLa cells were infected with E. coli pInv1914 expressing invasin (MOI 100) for 6 h and luciferase assays were performed. Means + SD of two independent experiments are shown. (B) HeLa cells were transfected with pNF-βB Luc and pCMV-ß-Gal and co-transfected with pHM6, pHM6-YopT or pHM6-YopTC139A for 24 h. Subsequently HeLa cells were left untreated or infected with E. coli pInv1914 expressing invasin (MOI 100) for 6 h and luciferase assays were performed. Results of A and B are expressed as fold induction compared to uninfected cells treated with empty vector control and represent the mean + SEM of a representative experiment performed in quadruplicates. Asterisks indicate a significant difference between NF-κB activation of cells transfected with pHM6 compared to cells transfected with pHM6-YopT (p<0.05). (C, D) HeLa cells were transfected with siGILZ and cultured for 24 h and subsequently transfected with pHM6 or pHM6-YopT for additional 24 h. Cell lysates were harvested to determine GILZ and β-actin expression by immunoblot. NF-κB driven luciferase activity was assayed described for B. Means + SEM of three independent experiments. (TIF) [file pone.0040730.s001.tif]

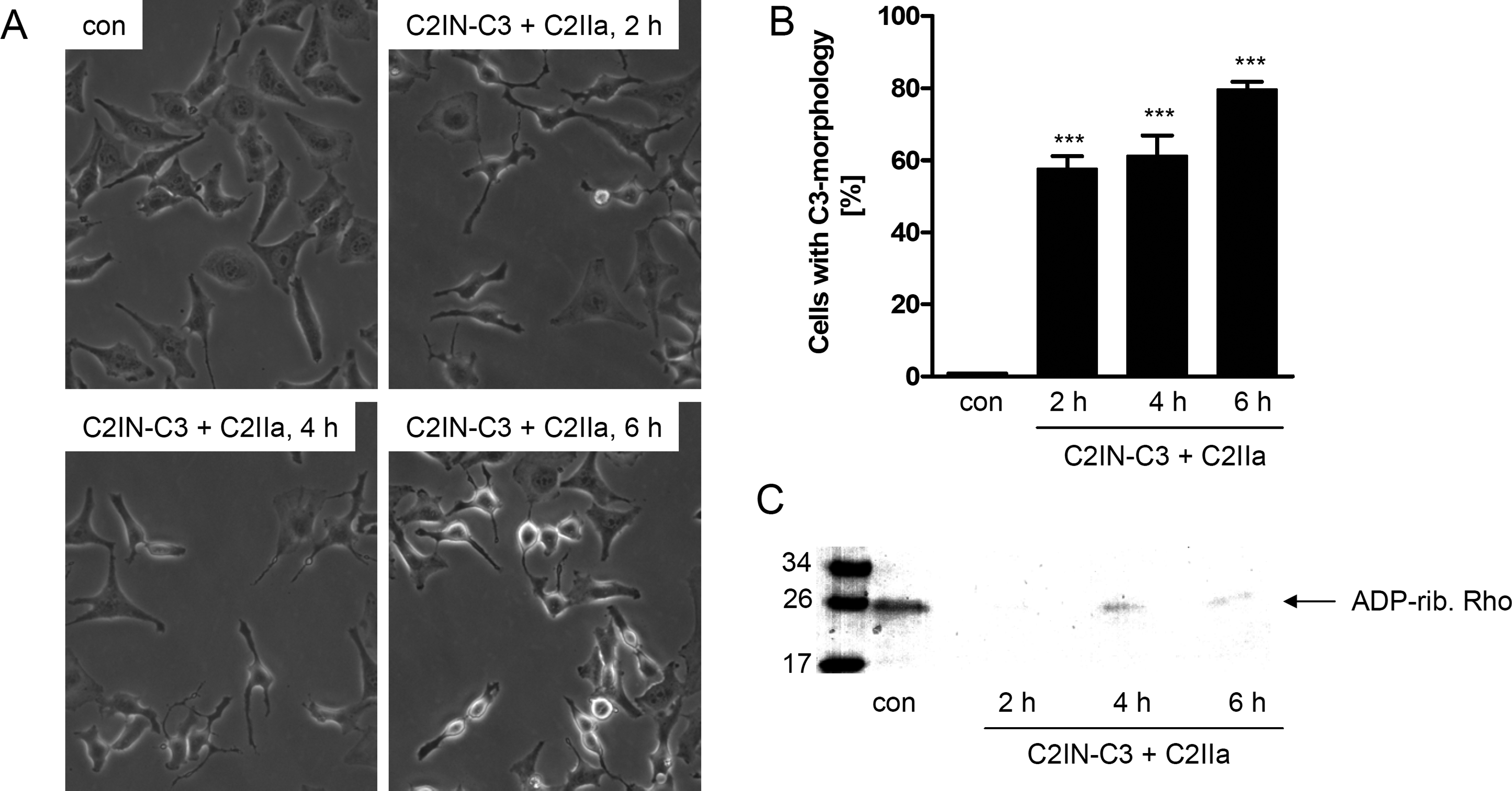

Supplement: Figure S2 — HeLa cell intoxication by C3 toxin Rho ADP-ribosylation. HeLa cells were incubated at 37°C with C2IN-C3lim (100 ng/mL) + C2IIa (200 ng/mL). After 2, 4 and 6 h pictures were taken to demonstrate the C3-induced change in cell morphology (A) and the cells were lysed. B. The percentages of cells showing “C3-morphology” were calculated from the pictures. Values are given as mean ± S.D. (n = 3); ** p<0.005. C. The ADP-ribosylation status of Rho from the cells was determined by sequential ADP-ribosylation. To this end, the cell lysates were incubated for 20 min at 37°C with biotin-labelled NAD+ and C2IN-C3lim (100 ng/mL). The proteins were separated by SDS-PAGE, blotted onto nitrocellulose and the biotin-labelled, i.e. ADP-ribosylated Rho was detected with streptavidin-peroxidase by Western blotting. The ADP-ribosylated Rho is shown. Comparable amounts of blotted lysate proteins were confirmed by Ponceau S-staining (not shown). (Note: In this experimental setting unlabeled Rho ADP-ribosylation in the intact cells competes with biotin-labelled ADP-ribosylation after lysis. A strong signal therefore means that Rho was not ADP-ribosylated in the intact cells, a weak signal indicates ADP-ribosylation of Rho by the toxin in the intact cells prior to lysis). (TIF) [file pone.0040730.s002.tif]

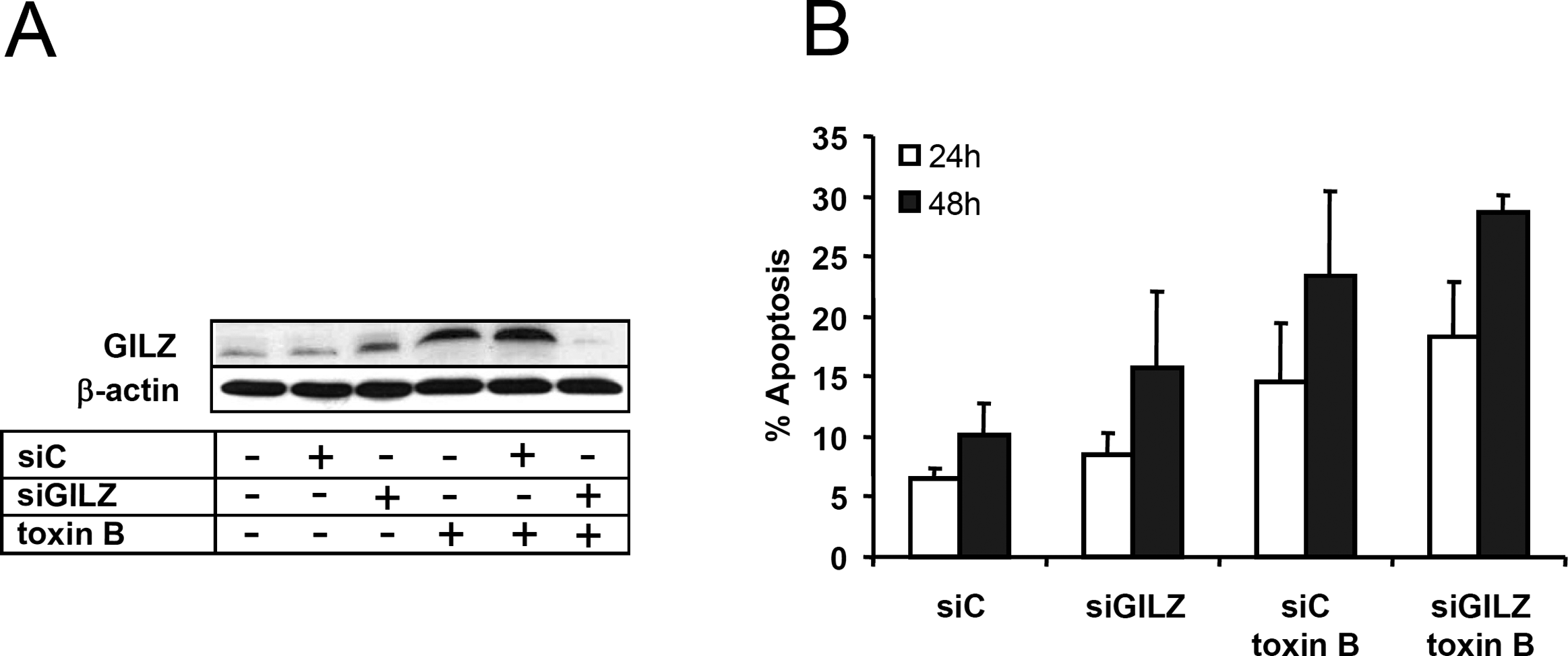

Supplement: Figure S3 — Impact of GILZ on toxin B triggered apoptosis. HeLa cells were transfected with siGILZ for 48 h (A) and subsequently stimulated with Toxin B for additional 24 h or 48 h. Apoptotic cells were detected by Nicoletti assay. Results are expressed as mean + SEM of three independent experiments (B). (TIF) [file pone.0040730.s003.tif]
